# Expression Profiling and Functional Analysis of Candidate *Col10a1* Regulators Identified by the TRAP Program

**DOI:** 10.3389/fgene.2021.683939

**Published:** 2021-07-02

**Authors:** Huiqin Bian, Ting Zhu, Yuting Liang, Ruoxuan Hei, Xiaojing Zhang, Xiaochen Li, Jinnan Chen, Yaojuan Lu, Junxia Gu, Longwei Qiao, Qiping Zheng

**Affiliations:** ^1^Department of Hematology and Hematological Laboratory Science, Jiangsu Key Laboratory of Medical Science and Laboratory Medicine, School of Medicine, Jiangsu University, Zhenjiang, China; ^2^Laboratory of Clinical Medicine, Huai'an Women & Children Hospital, Affiliated to Yangzhou University, Huai'an, China; ^3^Center of Clinical Laboratory, The First Affiliated Hospital of Soochow University, Suzhou, China; ^4^Shenzhen Academy of Peptide Targeting Technology at Pingshan and Shenzhen Tyercan Bio-Pharm Co., Ltd., Shenzhen, China; ^5^Suzhou Affiliated to State Key Laboratory of Reproductive Medicine, School of Gusu, The Affiliated Suzhou Hospital of Nanjing Medical University, Nanjing Medical University, Suzhou, China

**Keywords:** *Col10a1* regulators, Tbx-5, Runx2, TRAP program, chondrocyte hypertrophy, skeletal disease

## Abstract

Hypertrophic chondrocytes and their specific marker, the type X collagen gene (*Col10a1*), are critical components of endochondral bone formation during skeletal development. We previously found that Runx2 is an indispensable mouse *Col10a1* gene regulator and identified many other transcription factors (TFs) that potentially interact with the 150-bp *Col10a1* cis-enhancer. However, the roles of these candidate TFs in *Col10a1* expression and chondrocyte hypertrophy have not been elucidated. Here, we focus on 32 candidate TFs recently identified by analyzing the 150-bp *Col10a1* enhancer using the transcription factor affinity prediction (TRAP) program. We found that 12 TFs (Hoxa3, Lsx, Evx2, Dlx5, S8, Pax2, Egr2, Mef2a, Barhl2, GKlf, Sox17, and Crx) were significantly upregulated and four TFs (Lhx4, Tbx5, Mef2c, and Hb9) were significantly downregulated in hypertrophic MCT cells, which show upregulation of *Col10a1* expression. Most of the differential expression pattern of these TFs conformed with the results obtained from ATDC5 cell model and primary mouse chondrocytes. Notably, *Tbx5* was downregulated upon *Col10a1* upregulation, overexpression of *Tbx5* decreased *Col10a1* expression, and knock-down of *Tbx5* increased *Col10a1* expression in hypertrophic chondrocytes, suggesting that Tbx5 is a negative regulator of *Col10a1*. We further generated a stable *Tbx5*-overexpressing ATDC5 cell line and *ColX-Tbx5* transgenic mice driven by *Col10a1*-specific enhancers and promoters. *Tbx5* overexpression decreased *Col10a1* expression in ATDC5 cells cultured as early as day 7 and in limb tissue on post-natal day 1. Slightly weaker alkaline phosphatase staining was also observed in cell culture on day 7 and in limb digits on embryonic day 17.5, indicating mildly delayed ossification. Further characterization of these candidate *Col10a1* transcriptional regulators could help identify novel therapeutic targets for skeletal diseases associated with abnormal chondrocyte hypertrophy.

## Introduction

Endochondral ossification is a major developmental pathway for most of the appendicular skeleton (i.e., long bones) and some of the axial skeleton (i.e., flat bones). Hypertrophic chondrocytes and their specific marker, the type X collagen gene (*Col10a1*), are two critical components of the endochondral pathway during long bone development (Mackie et al., 2008; Debiais-Thibaud et al., 2019). Although present at a late stage of chondrocyte differentiation, hypertrophic chondrocytes are implicated as the principal engine of bone growth, largely due to their association with blood vessel invasion and calcified matrix deposition, which are critical for endochondral ossification (Linsenmayer et al., 1991). Type X collagen also facilitates endochondral ossification by affecting hematopoiesis and promoting matrix mineralization (Shen, 2005; Grskovic et al., 2012). Mutant or abnormal human *COL10A1* expression are often accompanied by abnormal chondrocyte hypertrophy, as seen in children and young adults with multiple skeletal dysplasia (Warman et al., 1993; Ikegawa et al., 1998; Bateman et al., 2005; Lu et al., 2014; Ain et al., 2018). Abnormal *COL10A1* expression and chondrocyte hypertrophy are also observed in elderly individuals with osteoarthritis (OA), who show osteophyte formation involving a process mimicking the endochondral pathway (von der Mark et al., 1995; Girkontaite et al., 1996; Drissi et al., 2005; Lamas et al., 2010; Saito et al., 2010; Armiento et al., 2019; Gratal et al., 2019; He et al., 2019). Recent studies show that intact trimeric noncolla-genous 1 domain of type X collagen is a degradation by-product of endochondral ossification released into the circulation in proportion to the overall growth plate. Thus, its detection may be useful for monitoring growth in the pediatric population, fracture healing, scoliosis, arthritis, and cancer (Coghlan et al., 2017). Together, these findings indicate the existence of close relationships among *Col10a1* expression, chondrocyte hypertrophy, and endochondral ossification. Therefore, regulators of hypertrophic chondrocyte-specific *Col10a1* expression may play essential roles in chondrocyte hypertrophy.

Multiple transcription factors (TFs) and signaling pathways regulate hypertrophic chondrocyte-specific *Col10a1* expression *in vitro* and *in vivo* (Lu et al., 2014). Of these, Runx2 is essential for osteoblast differentiation and chondrocyte hypertrophy (Komori et al., 1997; Lee et al., 1997; Otto et al., 1997; Komori, 2018; Liao et al., 2019; Qin et al., 2020) and is a major transcriptional determinant for *Col10a1* expression across species (Drissi et al., 2003; Simoes et al., 2006; Higashikawa et al., 2009). In our work on mouse *Col10a1* regulation, we found that Runx2 directly interacts with the *Col10a1* proximal promoter and 150-bp enhancer and is an indispensable *Col10a1* regulator (Zheng et al., 2003, 2009; Li et al., 2011). However, we also found that additional TFs and/or co-factors are required for cell-specific *Col10a1*/reporter expression *in vivo* (Li et al., 2011). Most recently, using combined bioinformatics and proteomic approaches, we identified many TFs that may interact with the 150-bp *Col10a1* cis-enhancer (Gu et al., 2014). Of these, more than 50 candidate TFs were identified by the transcription factor affinity prediction (TRAP) program (Kel et al., 2003; Thomas-Chollier et al., 2011; Gu et al., 2014). However, how these candidate TFs regulate cell-specific *Col10a1* expression and affect chondrocyte hypertrophy remain largely unknown.

Here, we systematically examined the expression levels of candidate TFs in *in vitro* and *ex vivo* chondrogenic cell models showing increased *Col10a1* expression upon hypertrophy. From these candidate TFs, we identified many potential *Col10a1* transactivators and repressors that promote or inhibit chondrocyte hypertrophy and are associated with skeletal diseases. We specifically investigated the role of Tbx5, a potential candidate Col10a1 repressor, during endochondral ossification using two mouse chondrogenic cell models: the MCT and ATDC5 cell lines. In addition, we generated *ColX-Tbx5* transgenic mice with specific expression of exogenous *Tbx5* gene in hypertrophic chondrocytes to study the effect of Tbx5 on *Col10a1* expression and endochondral ossification *in vivo*. Our findings suggest that these candidate *Col10a1* transcriptional regulators, including Tbx5, are potential therapeutic targets in collagenopathy and skeletal diseases associated with abnormal *Col10a1* expression and chondrocyte hypertrophy.

## Results

### Candidate TF Binding Sites Within the *Col10a1* Cis-Enhancer

**Figure 1:**
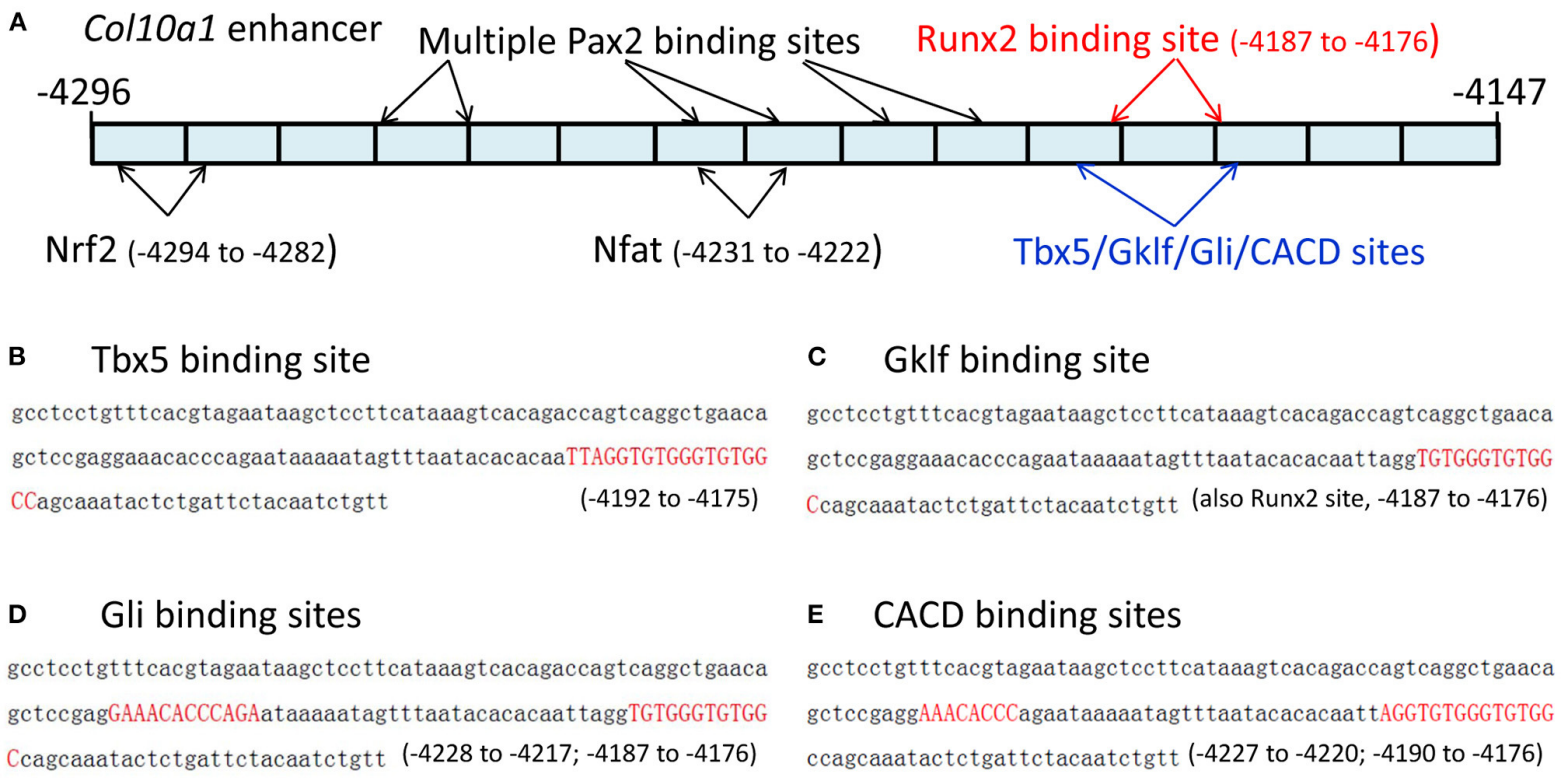
The 150-bp *Col10a1* cis-enhancer and its binding sites for selected candidate TFs. **(A)** Schematic illustration of the cis-enhancer derived from the *Col10a1* distal promoter region (−4,296 to −4,147 bp). Positions of putative binding sites for Pax2, Nrf2, Nfat, Tbx5, Gklf, Gli, CACD, and Runx2 are shown. **(B–E)** Sequences of predicted binding sites for Tbx5, Gklf, Gli, and CACD are highlighted in red. These TFBSs are the same as or overlap with the previously described Runx2 binding site TGTGGGTGTGGC (−4,187 to −4,176 bp) (Li et al., 2011).

We recently reported the *in silico* sequence analysis of the 150-bp *Col10a1* cis-enhancer to search for TF binding sites (TFBSs) using multiple web-based programs (Gu et al., 2014). Here, we provide detailed information on these candidate TFs identified by the TRAP program (Kel et al., 2003; Thomas-Chollier et al., 2011; Gu et al., 2014). We identified 48 potential binding sites for candidate TFs that showed a *p*-value < 0.05, and were ranked from lowest to highest *p*-value based on their binding affinity (Supplementary Figure 1). The search criteria were set for mouse promoters (background model) in the transfac_2010.1 vertebrate (matrix file) database. These predicted 48 TFBSs represented ~40 candidate TFs, including multiple MEF2 and Tbx5 sites (Supplementary Figure 1) with slight sequence differences. These candidate TFs included homeobox-containing genes (Hoxa3, Hoxa4, Hoxa13, Lhx4, Lhx8, Lhx61), a zinc finger gene (Gklf), mouse embryonic fibroblast markers, paired box (Pax) family members (Pax2, Pax7), and Tbx5. Both 5′- and 3′-primer ends of the enhancer showed multiple TFBSs, although more potential TFBSs were found at the 3′-end, which is the location of the previously described putative Runx2 binding site (Figure 1A) (Gu et al., 2014). Specifically, Tbx5, CACD, Gklf, EGR2, and Gli bind to a sequence that is the same or similar to the previously described Runx2 binding site (Li et al., 2011). Hox3a, Brahl1, Brahl2, Lhx8, Isx, Lhx4, Lhx61, Evs2, Pou6f1, Gbx1, Pax7, Dlx5, S8, Brax2, Hb9, and Hoxa4 bind to a sequence adjacent to the Runx2 site (Figures 1B–E, data not shown). Six putative TFs (Og2, CACD, Tbx5, Tst-1, Rsrfc4, and Foxj2) were predicted to bind to the cis-enhancer using the MATCH program by searching the TRANSFAC database when the score was increased to 90. Intriguingly, all putative TFs were identified by both the TRAP and MATCH programs.

### *Col10a1* and Candidate TF Gene Expression in Chondrogenic Cell Models

**Figure 2:**
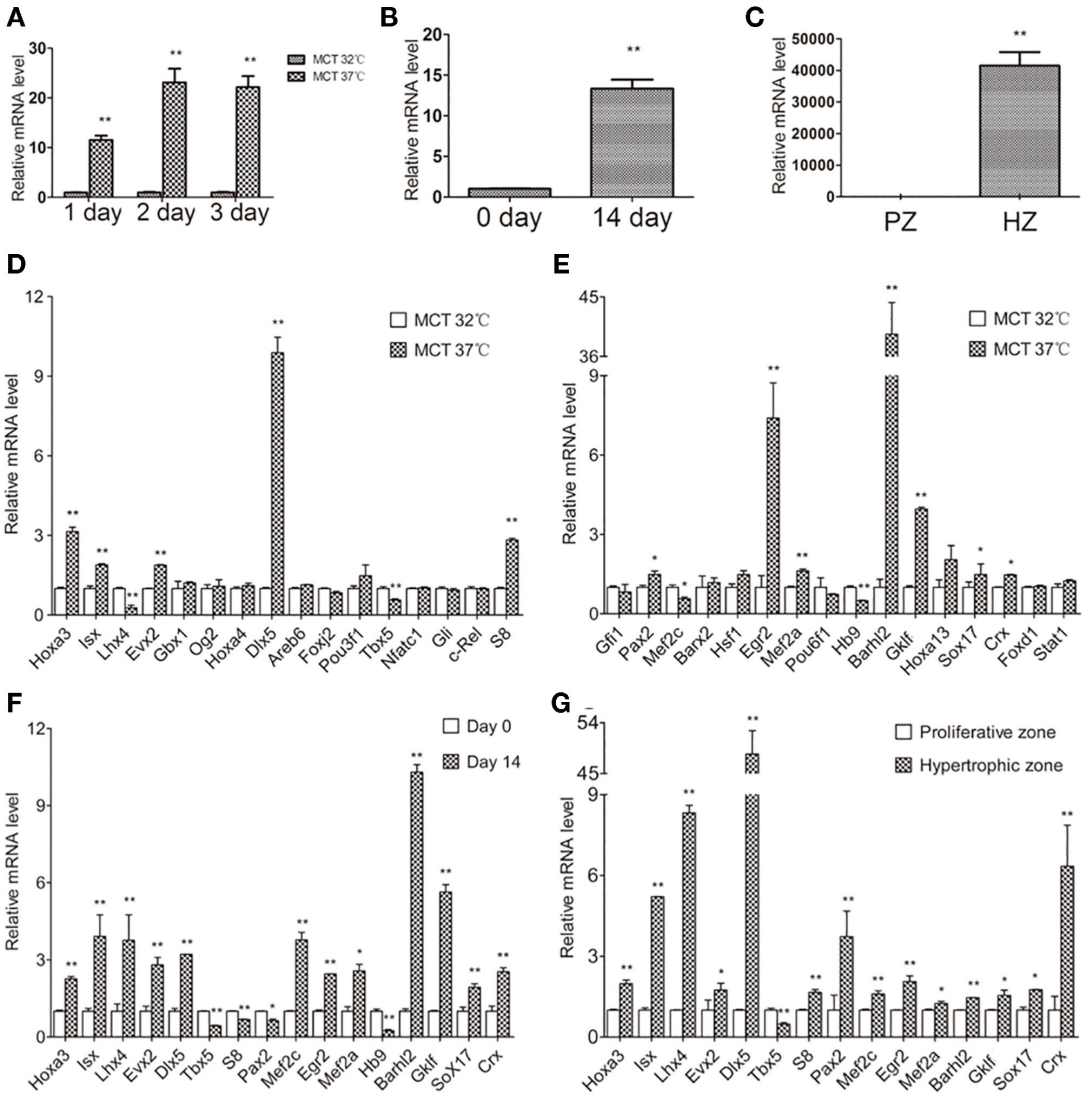
Relative *Col10a1* and candidate TFs mRNA levels in hypertrophic vs. proliferative chondrocytes. **(A)** *Col10a1* mRNA levels were upregulated in hypertrophic compared with proliferative MCT cells at 1, 2, and 3 days. **(B)** *Col10a1* mRNA level in ATDC5 cells was upregulated on day 14 compared with day 0. **(C)** *Col10a1* mRNA was barely detectable in proliferative primary chondrocytes but was abundantly expressed in hypertrophic primary chondrocytes. **(D,E)** *Hoxa3, lsx, Evx2, Dlx5, S8, Pax2, Egr2, Mef2a, Barhl2, GKlf, Sox17*, and *Crx* were upregulated and *Lhx4, Tbx5, Mef2c*, and *Hb9* were downregulated in hypertrophic MCT cells. **(F)** *Hoxa3, lsx, Lhx4, Evx2, Dlx5, Egr2, Mef2a, Mef2c, Barhl2, GKlf, Sox17*, and *Crx* were upregulated and *S8, Pax2, Tbx5*, and *Hb9* were downregulated in hypertrophic ATDC5 cells. **(G)** Except for *Tbx5*, all other genes examined were significantly upregulated in hypertrophic primary chondrocytes. *p < 0.05, **p < 0.01.

Next, we examined *Col10a1* expression in MCT and ATDC5 cells as well as primary chondrocytes derived from mouse limbs and rib growth plates. MCT cells are mouse chondrocytes that show upregulated *Col10a1* expression when cultured under 37°C (i.e., hypertrophic) but not 32°C (i.e., proliferative) conditions (Gu et al., 2014). As expected, after culture for 1, 2, or 3 days at 37°C, *Col10a1* mRNA levels were upregulated by 11.6-fold (*p* = 0.0002), 23.1-fold (*p* = 0.0006), and 22.2-fold (*p* = 0.0006), respectively (Figure 2A). In addition, ATDC5 cells are an established model of endochondral ossification that show signifi**c**ant upregulation of *Col10a1* after culture for long periods of time (i.e., 14 days) and/or insulin, transferrin, and sodium selenite (ITS) induction (Shukunami et al., 1997; Gu et al., 2013). Indeed, compared with cells on day 0 without ITS induction, *Col10a1* expression was 13.3-fold higher (*p* = 0.0004) in hypertrophic ATDC5 cells maintained in ITS medium for 14 days (Figure 2B). Moreover, *Col10a1* was abundantly expressed in hypertrophic primary chondrocytes but was barely detectable in proliferative primary chondrocytes micro-dissected from corresponding zones of growth plates (Figure 2C). Together, these results demonstrate the marked upregulation of *Col10a1* in hypertrophic MCT cells, ATDC5 cells, and primary chondrocytes compared with their corresponding proliferative chondrocytes.

To determine their correlation with *Col10a1* expression, we systematically examined mRNA levels for all candidate TFs identified by the TRAP program (Supplementary Figure 1) in proliferative and hypertrophic MCT cells on day 3 (Figure 2A). We found that 12 candidate genes (*Hoxa3, lsx, Evx2, Dlx5, S8, Pax2, Egr2, Mef2a, Barhl2, GKlf, Sox17*, and *Crx*) were significantly upregulated and four candidate genes (*Lhx4, Tbx5, Mef2c*, and *Hb9*) were significantly downregulated in hypertrophic MCT cells (Figures 2D,E). To confirm the differential expression of candidate TFs in proliferative vs. hypertrophic cells, we examined their mRNA expression in ATDC5 cells with or without hypertrophic induction (primers see Table 1). Despite some discrepancies, most candidate genes were up- or downregulated in similar directions as those in MCT cells (Figure 2F, Table 2). We also performed expression analysis of differentially expressed candidate genes in proliferative vs. hypertrophic primary chondrocytes. Again, despite some discrepancies, the results were similar to those obtained in MCT and ATDC5 cells (Figure 2G, Table 2). Across all three hypertrophic cell models, *Hoxa3, lsx, Evx2, Dlx5, Egr2, Mef2a, Barhl2, GKlf, Sox17*, and *Crx* were significantly upregulated, whereas *Tbx5* and *Hb9* were significantly downregulated or undetectable. Thus, the differential expression of these candidate genes upon *Col10a1* upregulation in hypertrophic cell models suggests their distinct roles in regulating *Col10a1* expression.

**Table 1:** The primers in real-time qRT-PCR.

	**RefSeqID**	**Sense primer (5^**′**^-3^**′**^)**	**Antisense primer (5^**′**^-3^**′**^)**	**Amplicon (bp)**
*Hoxa3*	NM_010452.3	TTCCACTTCAACCGCTACCT	TTCTTGTACTTCATGCGGCG	116
*Isx*	NM_027837.3	GCAATCCTGAAGAAACCCACA	AACCTGGGATAGTTGTCTGC	91
*Lhx4*	NM_010712.2	GAGACAGCCAAGCAAAACGA	TGGGGAGTTCTTGTATGCGT	108
*Evx2*	NM_007967.2	AAGCACCGTCTCCTCCGAA	CCACGTCGCTGCTCATGTC	101
*Gbx1*	NM_015739.2	AGTGAGGTGCAGGTGAAGAT	TATGGGCACTACAATCTTGG	123
*Og2*	NM_130869.3	TGACAGTGACAAACGCCATG	TTTTCTCCACTTTGCCCTGC	97
*Hoxa4*	NM_008265.3	TTCCACTTTAACCGCTACCTG	TCTTTCTTCCACTTCATTCTCCG	119
*Dlx5*	NM_010056.3	ACCCGTCTCAGGAATCGCCAA	TTTGCCATAAGAAGCAGAGGTAG	119
*Areb6*	NM_011546.3	GTCACTGATGTTCCTCCCCA	GAGGCAAAACAGTGAGCACA	137
*Foxj2*	NM_021899.3	CCTCATCAGCACCATCCCC	TCAATATTGGAGCACCAGTCA	122
*Pou3f1*	NM_011141.2	CAAATTTGGGGTGAGGTGGG	TAGGATGGGGAGGGAGAACA	127
*Tbx5*	NM_011537.3	CCCCACCTAACCCATACCC	GATGTCTCCATGTACGGCTTC	121
*Nfatc1*	NM_016791.4	AGATCCCGTTGCTTCCAGAA	CTCCCCTTTCCTCAGCTCAA	98
*Gli*	AF026305.1	TCAGCTGGACTTTGTGGCTA	CAGAGGGAGATGGGGTGTTT	98
*c-Rel*	NM_009044.2	CTTCACAACTGCTCTGCCTC	CAGTTCTTGTTCACACGGCA	99
*S8*	NM_009116.2	ATCTATCCTGGCCAGCATCC	GTTGGCCATGTTGACTCCAG	98
*Gfi1*	NM_001267621.1	CGAAGCCCAGCCCTACACG	CGCTGCACTGCCGATAGCTC	101
*Pax2*	NM_011037.4	ATGACGAGCACCACTCTACCTG	TGCCTGAGAACTCGCTCCC	112
*Mef2c*	NM_001170537.1	TCAGTTGGGAGCTTGCACTA	TGGTGGTACGGTCTCTAGGA	119
*Barx2*	NM_013800.2	TGATACCCAGGAGCCCAAAG	CCCCTTCCCCTCAAAGAACT	134
*Hsf1*	NM_008296.2	CTAACCAAGCTGTGGACCCTC	TCAATGTGGACTACTTTTCGG	200
*Egr2*	NM_010118.3	GCCCCTTTGACCAGATGAACG	TGCCCATGTAAGTGAAGGTCT	147
*Mef2a*	NM_001033713.1	GGGGTGACTTCCATTCTCCA	CATGTGTCCATCCTCATGCG	94
*Pou6f1*	NM_010127.3	CCTATCCAGCCGACACAAGC	TCTTCTAAGTTGATCCCGTCCT	182
*Hb9*	AF153046.1	CTCGCCTCCTCCAAGACTAG	TAGCCATCTTTCGCATCCCT	91
*Barhl2*	NM_001005477.1	TTCTCCTCATCACACCCCGAA	CCTCCTTTGTTCCGTGGCAT	143
*GKlf*	NM_010637.3	AACTACCCTCCTTTCCTGCC	CACGACCTTCTTCCCCTCTT	125
*Hoxa13*	NM_008264.1	ATGACAGCCTCCGTGCTCC	CGCCCCTTCCATGTTCTTGTTG	114
*Sox17*	NM_011441.4	GTTGACCTTGGCAGAGAAGC	CCGGTACTTGTAGTTGGGGT	91
*Crx*	NM_007770.4	TCTCAGCAAGCAACAGCAAG	TGCTGTAAAGGGGCTAAGCT	106
*Foxd1*	NM_008242.2	CTCATCACCATGGCCATCCT	GGTTGTGACGGATGCTGTTC	133
*Stat1*	NM_001205313.1	CATGGCTGCCGAGAACATAC	AGTTCGCTTAGGGTCGTCAA	139
*Col10a1*	NM_009925.4	TCTGTGAGCTCCATGATTGC	GCAGCATTACGACCCAAGATC	201
*Gapdh*	NM_008084.2	CACATTGGGGGTAGGAACAC	ACCCAGAAGACTGTGGATGG	171

**Table 2:** Fold changes in mRNA levels of candidate TFs in hypertrophic vs. proliferative chondrocytes.

	**MCT (37 vs. 32**^****°****^**C)**		**ATDC5 (day 14 vs. day 0)**		**Mouse HZ vs. PZ**	
	**Fold change**	***p*-value**	**Fold change**	***p*-value**	**Fold change**	***p*-value**
Hoxa3	3.14↑	0.0003	2.25↑	0.0003	1.97↑	0.0030
Isx	1.88↑	0.0024	3.91↑	0.0137	5.20↑	0.0005
Lhx4	0.27↓	0.0038	3.75↑	0.0409	8.31↑	0.0016
Evx2	1.87↑	0.0009	2.80↑	0.0096	1.74↑	0.0397
Dlx5	9.88↑	0.0067	3.21↑	0.0022	48.4↑	0.0003
Tbx5	0.56↓	0.0370	0.43↓	7.59E05	0.47↓	0.0072
S8	2.82↑	3.36E05	0.68↓	0.0002	1.65↑	0.0031
Pax2	1.48↑	0.0264	0.63↓	0.004331	3.72↑	0.0106
Mef2c	0.56↓	0.0102	3.77↑	0.0007	1.59↑	0.0072
Egr2	7.39↑	0.0020	2.44↑	0.001517	2.05↑	0.0067
Mef2a	1.61↑	0.0014	2.56↑	0.0029	1.24↑	0.0474
Hb9	0.49↓	0.0009	0.24↓	0.0029	—	—
Barhl2	39.35↑	0.0004	10.2↑	7.26E06	1.46↑	0.0024
GKlf	3.95↑	5.30E06	5.6↑	0.0004	1.54↑	0.0423
Sox17	1.47↑	0.0373	1.93↑	0.0085	1.75↑	0.0352
Crx	1.46↑	0.0153	2.53↑	0.0026	6.33↑	0.0204

### *Col10a1* and Candidate TF Protein Expression in MCT Cells and Growth Plate Chondrocytes

**Figure 3:**
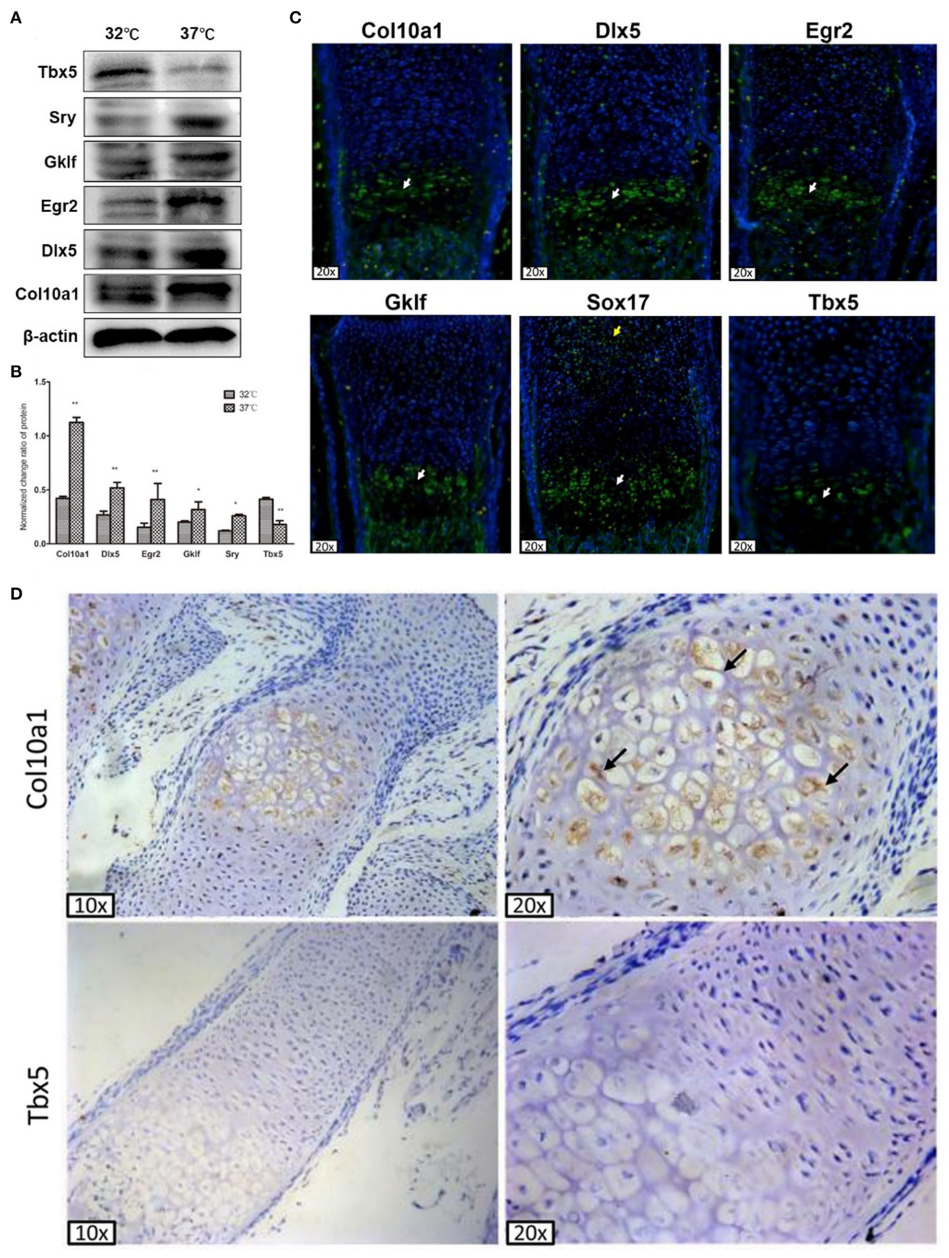
Col10a1 and candidate TFs protein expression in hypertrophic chondrocytes. **(A)** Protein levels of *Col10a1, Dlx5, Egr2, Klf4*, and *Sox17* were increased and that of *Tbx5* was decreased in hypertrophic compared with proliferative MCT cells. **(B)** Relative protein levels of *Col10a1, Dlx5, Egr2, Gklf*, *Sox17*, and *Tbx5* normalized to β-actin in hypertrophic vs. proliferative MCT cells. **(C)** Strong green fluorescence signal indicating Col10a1 protein expression was observed throughout the hypertrophic zone. Dlx5, Egr2, Gklf, and Sox17 protein were also abundantly expressed in hypertrophic chondrocytes, with some Sox17 signal also seen in resting or proliferative chondrocytes. Tbx5 signal was much weaker in hypertrophic chondrocytes compared with that of other TFs. **(D)** Immunohistochemistry analysis detected strong Col10a1 expression only in the extracellular matrix of hypertrophic chondrocytes (top row), whereas no obvious Tbx5 expression was detected in either proliferative or hypertrophic chondrocytes. *p < 0.05, **p < 0.01.

We performed western blot analysis to measure protein levels of Tbx5, Sox17, Gklf, Egr2, and Dlx5 in proliferative and hypertrophic MCT cells, as the mRNA levels of these TFs were consistently upregulated (*Sox17, Gklf, Egr2*, and *Dlx5*) or downregulated (*Tbx5*) in all three chondrogenic cell models. As expected, the amount of Col10a1 protein was significantly increased in hypertrophic MCT cells (Figure 3A). Also, the protein levels of Sox17, Klf4, Egr2, and Dlx5 were significantly increased and that of Tbx5 was significantly decreased in hypertrophic as compared with proliferative MCT cells (Figures 3A,B). These results show that the protein levels of candidate TFs correspond to their mRNA levels in chondrogenic cell models.

To determine the *in vivo* relevance of these candidate genes to *Col10a1* expression and chondrocyte hypertrophy, we performed fluorescence immunohistochemistry of selected candidate TFs to measure their protein expression within growth plate (i.e., hypertrophic) chondrocytes. Sagittal sections of the distal radius were prepared for fluorescence immunostaining. As expected, Col10a1 protein was expressed throughout the hypertrophic zone as indicated by green fluorescence signal. Also, Dlx5, Egr2, Gklf, and Sox17 protein were abundantly expressed in hypertrophic chondrocytes, with only Sox17 expressed in resting or proliferative chondrocytes (Figure 3C). We also detected fluorescence signal for Tbx5 in hypertrophic chondrocytes, but its intensity was weaker than that of the other TFs (Figure 3C). These results demonstrate the high levels of expression of most candidate TFs in hypertrophic chondrocytes, consistent with their high levels of mRNA and protein expression in hypertrophic MCT cells (Figures 2D–G, 3A).

To measure Tbx5 expression in hypertrophic chondrocytes in relation to Col10a1 expression, we performed immunohistochemistry analysis using Tbx5 and Col10a1 antibodies in sagittal sections from the distal ulna and fibula. Col10a1 was abundantly expressed in the extracellular matrix of hypertrophic chondrocytes (Figure 3D, top right panel, black arrows). However, no obvious Tbx5 signal was detected in growth plate chondrocytes (Figure 3D, bottom panels). These results are consistent with the low expression of Tbx5 in hypertrophic chondrocytes and no obvious Tbx5 expression in resting or proliferative chondrocytes (Figures 3C,D).

### *Tbx5* Repression of *Col10a1* Expression in Endochondral Ossification Cell Models

**Figure 4:**
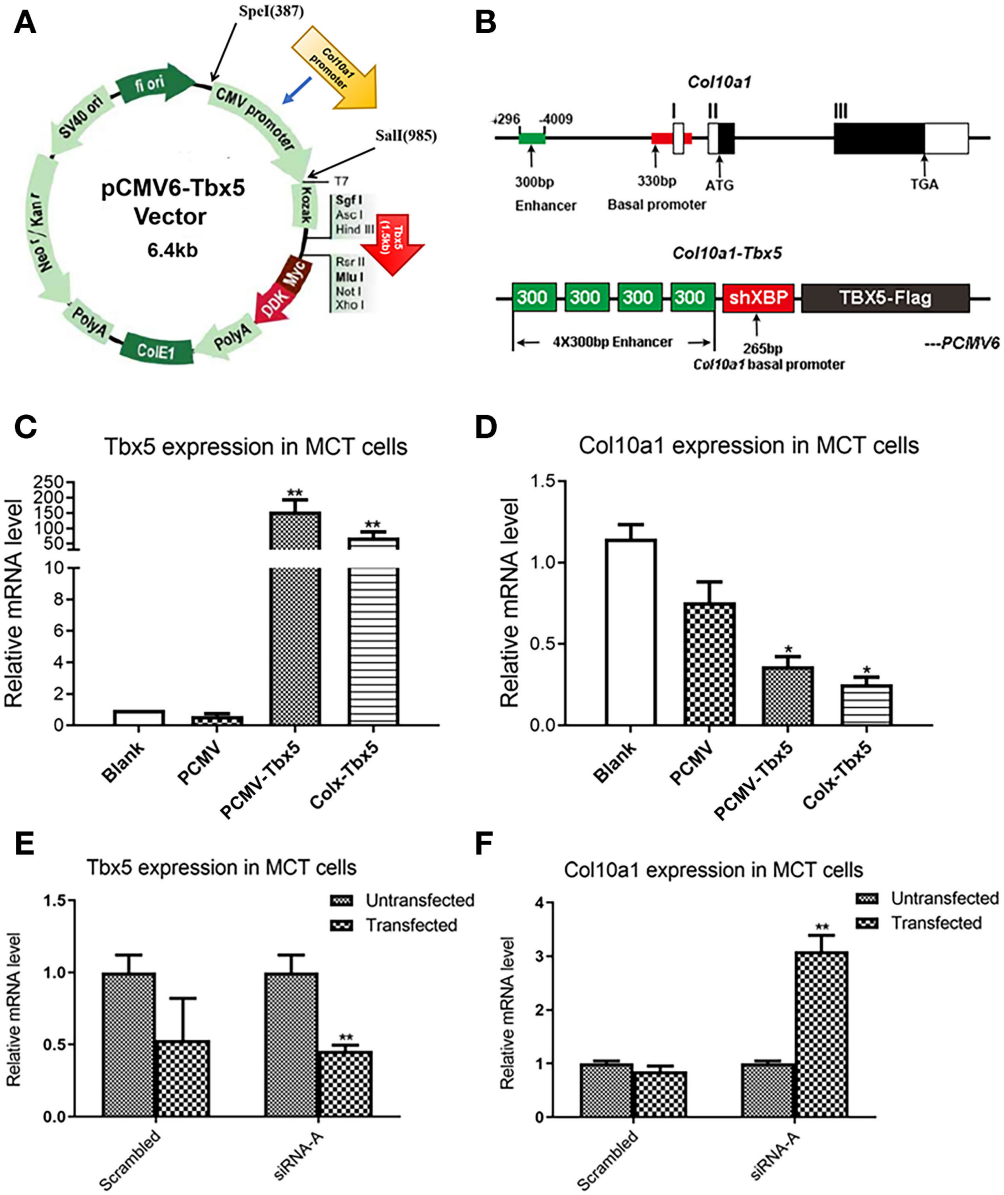
Transfection of *Tbx5* in MCT cells. **(A)** Schema graph of *Tbx5* expression plasmid driven by *Col10a1*-specific enhancer and promoter. **(B)** The *Col10a1* gene (top) and *Col10a1–Tbx5* transgenic construct (bottom). The ~300-bp hypertrophic chondrocyte-specific *Col10a1* cis-element illustrated previously locate in the distal promoter (−4,296 to −4,009 bp) and four copies of the ~300-bp cis-elements and a short basal promoter (265bp) element were used to drive the *Tbx5* gene with a Flag-tag. ATG: start codon; TAG: stop codon; ShXBP: Short *Col10a1* basal promoter. **(C,D)** Transient transfection of *Tbx5* increased the mRNA level of *Tbx5* and downregulated *Col10a1* expression. **(E,F)** Compared with scrambled RNA, *Tbx5* siRNA downregulated *Tbx5* expression and increased *Col10a1* expression. *p < 0.05, **p < 0.01.

We hypothesized that Tbx5 binds to a sequence of the *Col10a1* cis-enhancer that is also the binding site for Runx2, a known *Col10a1* transcriptional activator (Supplementary Figure 1, Figure 1). Interestingly, mRNA and protein levels of Tbx5 are significantly downregulated in hypertrophic MCT cells, ATDC5 cells, and primary chondrocytes (Figures 2, 3). To determine whether Tbx5 plays a repressive role in the regulation of *Col10a1* expression, we performed *in vitro* transient transfection of MCT cells using *Tbx5* expression plasmids and siRNAs. *Tbx5* expression plasmids were driven by the CMV and *Col10a1*-specific enhancer and promoter, respectively (Figures 4A,B). We found that overexpression of *pCMV*-*Tbx5* or *Col10a1*-*Tbx5* significantly increased Tbx5 expression (Figure 4C) but downregulated *Col10a1* expression (Figure 4D). By contrast, knocking down *Tbx5* in proliferative MCT cells significantly decreased Tbx5 expression (Figure 4E) increased *Col10a1* expression (Figure 4F). These results suggest an inverse correlation between *Tbx5* and *Col10a1* expression.

**Figure 5:**
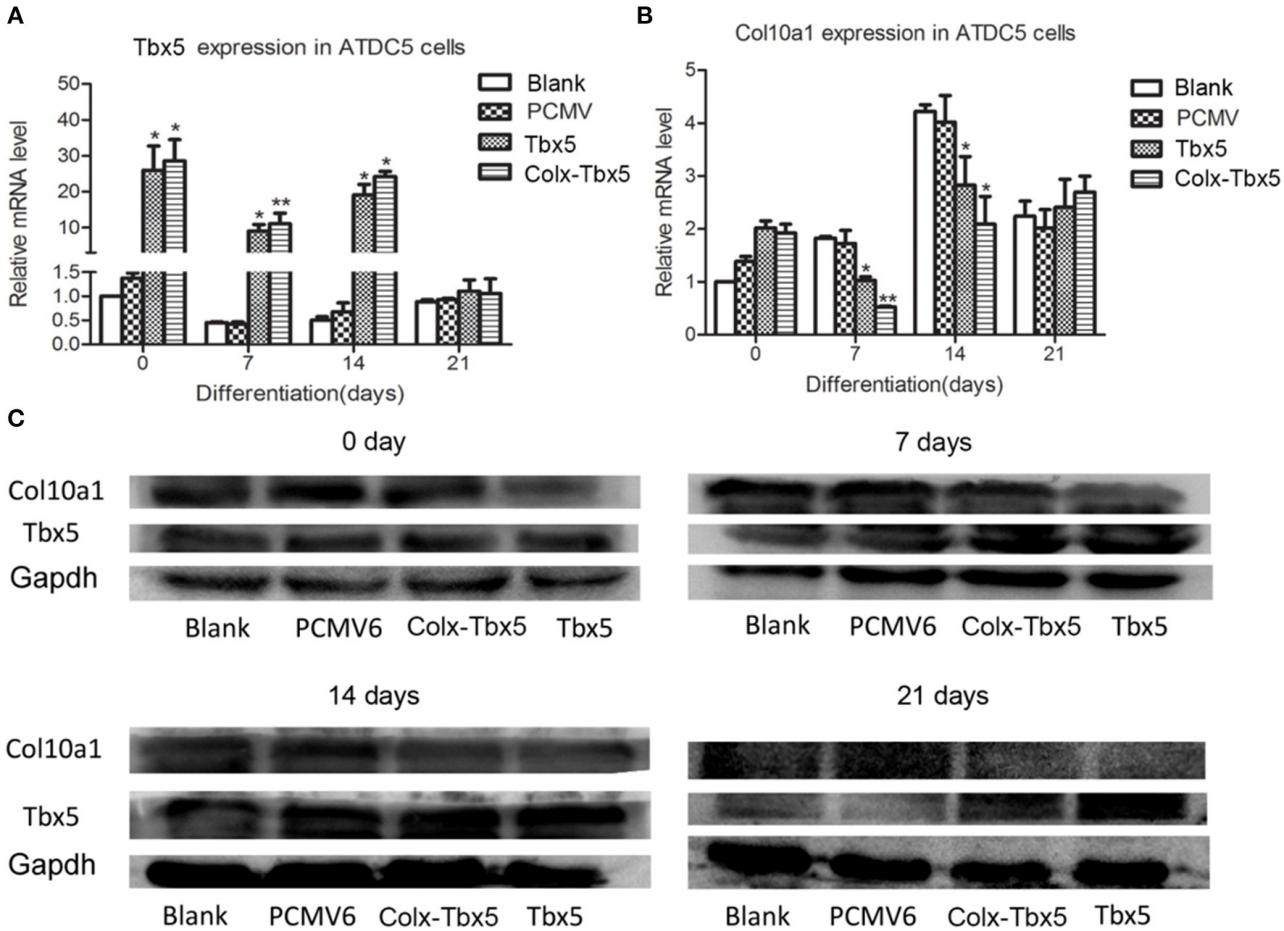
*Tbx5* overexpression inhibited *Col10a1* expression in ATDC5 cells. **(A)** *Tbx5* mRNA was upregulated in ATDC5 cells stably transfected with *Tbx5* expression plasmid compared with vector and blank controls. **(B)** *Col10a1* expression was inhibited in *Tbx5*-overexpressing cells compared with vector and blank controls. **(C)** Protein levels of Tbx5 and Col10a1 were similar to their mRNA levels. *p < 0.05, **p < 0.01.

We also generated a stable *Tbx5*-overexpressing ATDC5 cell line using *Tbx5*-expressing plasmid with *pCMV6*-entry control and G418 selection. Gene expression analysis showed significantly increased levels of *Tbx5* in ATDC5 cells stably transfected with *pCMV6-Tbx5* and *Colx-Tbx5* plasmid after 7 and 14 days of chondrogenic differentiation compared with blank and vector controls (Figure 5A). We also observed *Col10a1* expression in Tbx5-overexpressing cells after culture, with the highest level observed on day 14 (Figure 5B). Furthermore, the protein level of Col10a1 in *Tbx5*-overexpressing cells also peaked on day 14, consistent with its mRNA expression (Figure 5C). However, the protein level of Col10a1 in *Tbx5*-overexpressing cells was lower than that of controls on days 7, 14, and 21, in contrast to the protein level of Tbx5. These results demonstrate that overexpression of *Tbx5* inhibits Col10a1 expression in chondrogenic differentiation cell models.

### Effect of *Tbx5* Overexpression on Chondrogenic Differentiation in ATDC5 Cells

**Figure 6:**
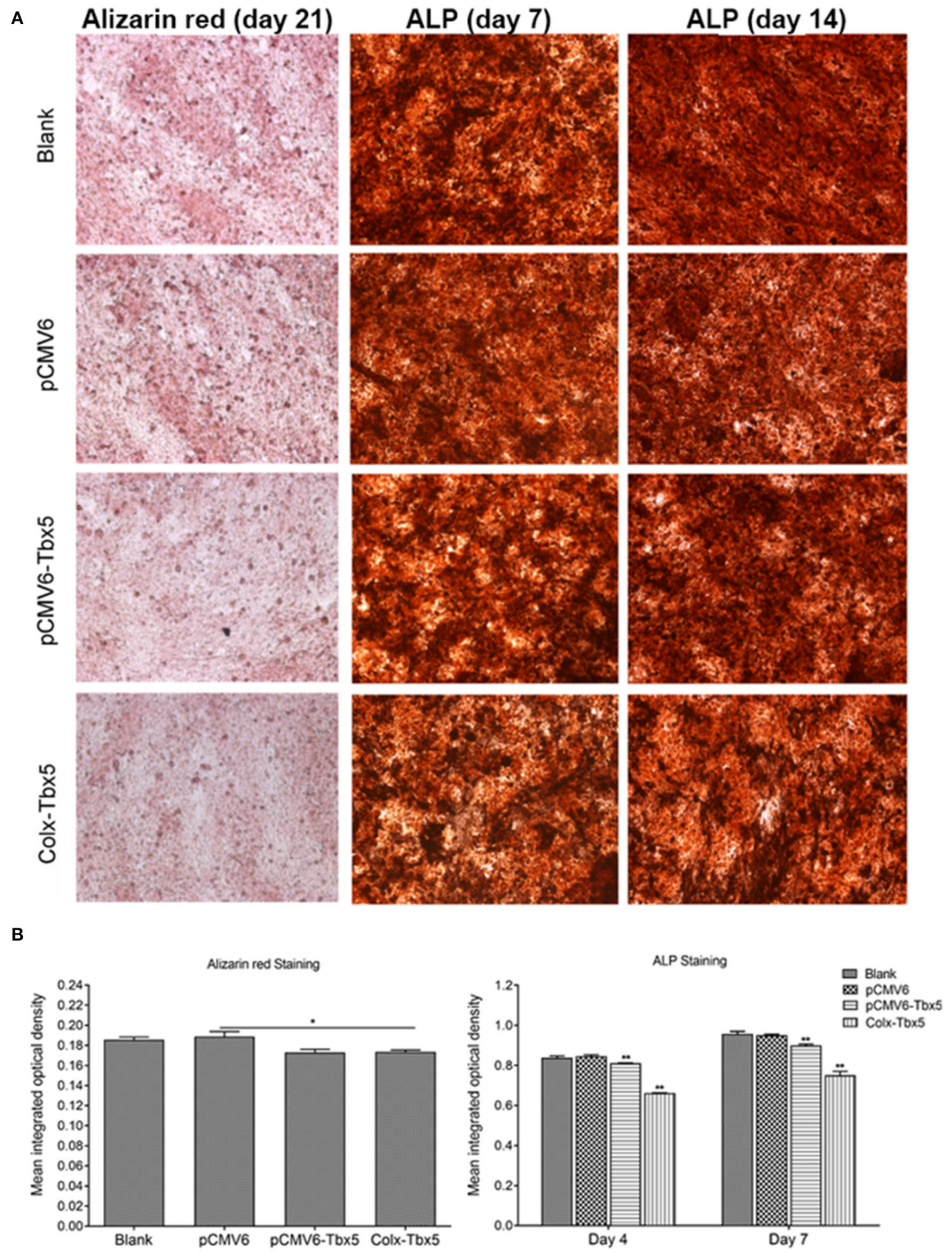
Effects of Tbx5 on chondrogenic differentiation in ATDC5 cells. **(A)** *Tbx5*-overexpressing cells showed slightly weaker Alizarin red staining than control cells on day 21 of culture. *Tbx5*-overexpressing cells showed slightly weaker ALP staining on days 4 and 7. **(B)** Quantification of mean integrated optical density of Alizarin red and ALP staining in *Tbx5*-overexpressing and control cells using Image-Pro Plus 6.0 image analysis software. Compared with control cells (blank, *pCMV6*), *Tbx5*-overexpressing cells (*Tbx5, ColX-Tbx5*) showed slightly weaker Alizarin red staining (left). *Tbx5*-overexpressing cells also showed slightly weaker ALP staining on days 4 and 7 (right). *p < 0.05, **p < 0.01.

To determine the effect of *Tbx5* overexpression on chondrogenic differentiation and mineralization in ATDC5 cells, we performed Alizarin red, Alcian blue, and alkaline phosphatase (ALP) staining. Alizarin red staining, indicative of cells reaching the late stage of *in vitro* ossification, was strongest on days 14 and 21. Compared with control cells (blank, *pCMV6*), staining was slightly weaker in *Tbx5*-overexpressing cells (*Tbx5, ColX-Tbx5*; Figures 6A,B), suggesting that Tbx5 plays a limited role at late stages of *in vitro* ossification in this cell model. No difference in Alcian blue staining was found between *Tbx5*-overexpressing and control cells on any day (Supplementary Figure 2), suggesting that Tbx5 has a limited effect on chondrocyte proliferation. Compared with controls, ALP staining was slightly weaker in *ColX-Tbx5* cells on days 4 and 7 (Figures 6A,B), suggesting that Tbx5 inhibits mineralization during chondrocyte maturation.

### *Tbx5* Overexpression Represses *Col10a1* Expression in *ColX-Tbx5* Transgenic Mice

**Figure 7:**
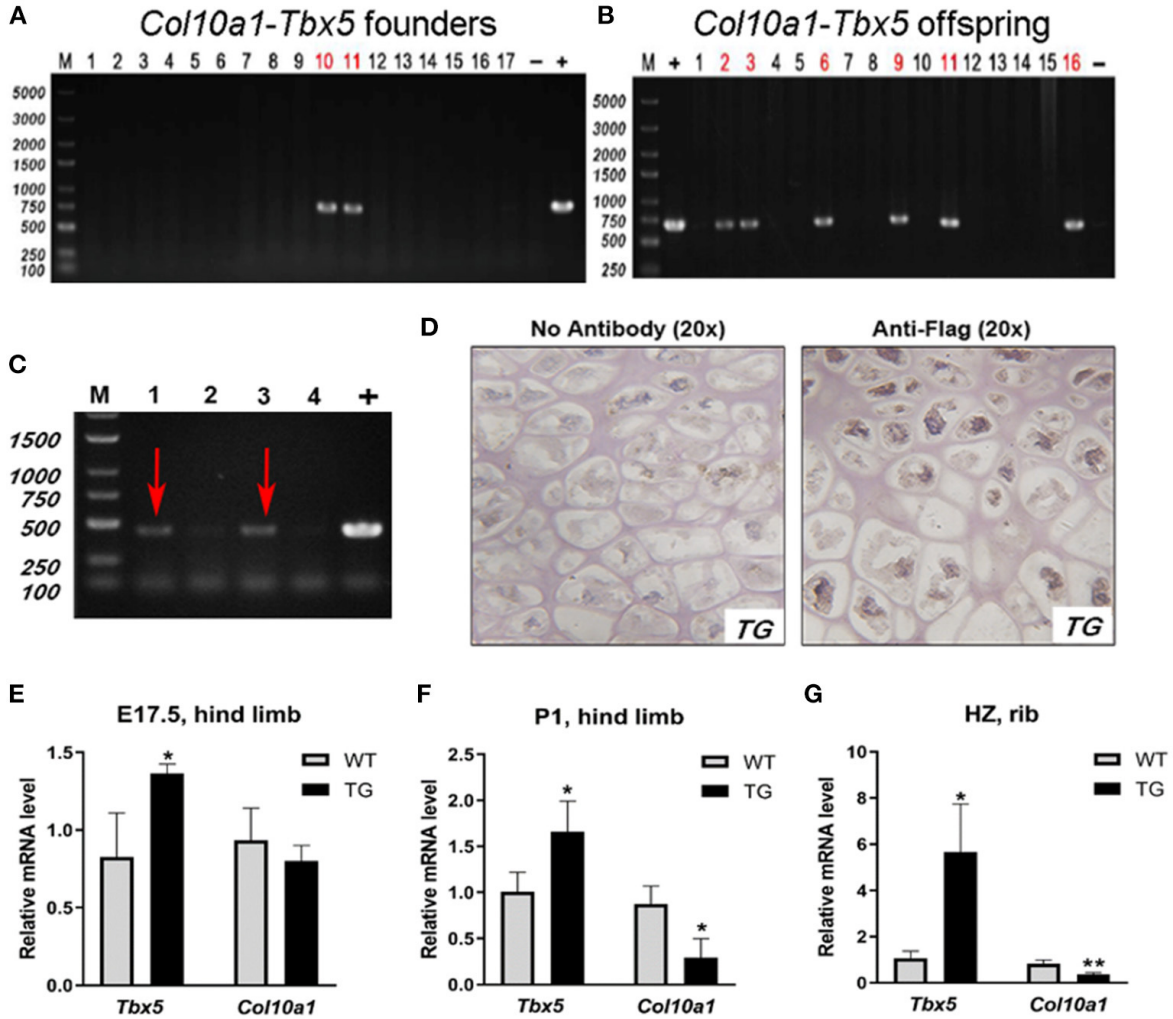
Expression of transgene and *Col10a1* mRNA in *Col10a1-Tbx5* TG mice. **(A)** *Col10a1-Tbx5* transgenic mouse lines. PCR genotyping using mouse skin genome DNA and *Col10a1-Tbx5* fragment specific primers indicated that we have successfully generated transgenic founders with ~10% positive rate (lanes 10, 11). **(B)** Genotype of the offspring of the transgenic founders breeding with wild-type mice was confirmed by PCR either and showed the establishment of *Col10a1–Tbx5* transgenic mouse lines. **(C)** RT-PCR confirmed transgene expression in *Col10a1-Tbx5* TG mice (red arrows). **(D)** Immunohistochemistry staining was used to analyze Flag expression in TG mouse hind limb sections. Dark brown staining shows Flag expression in hypertrophic chondrocytes of the proximal tibia in a TG mouse (right panel; control with no antibody, left panel). **(E–G)** *Tbx5* expression was upregulated in TG mice compared with WT mice at each age, whereas *Col10a1* expression was downregulated in limb tissue on P1 and the hypertrophic zone on P7. *p < 0.05, **p < 0.01.

To examine the putative function of *Tbx5* in regulating *Col10a1* expression in endochondral ossification *in vivo*, we established *ColX-Tbx5* transgenic (TG) mice with specific expression of exogenous *Tbx5* gene in hypertrophic chondrocytes using a microinjection strategy (Zheng et al., 2009) (Figures 7A,B). RT-PCR indicated that the transgene (Flag-tagged *Tbx5*) was expressed in TG mice but not in their wild-type (WT) littermates (Figure 7C). Tbx5 expression was specifically found in the nuclei of hypertrophic chondrocytes in the proximal tibia by immunohistochemistry staining with Flag antibody (Figure 7D). On embryonic day (E17.5), TG mice tended to show decreased *Col10a1* expression, but this change was not significant (Figure 7E). Compared with WT mice, TG mice showed significantly decreased *Col10a1* expression in limb tissue on post-natal day (P1) and the hypertrophic zone of ribs on P7 (Figures 7F,G).

**Figure 8:**
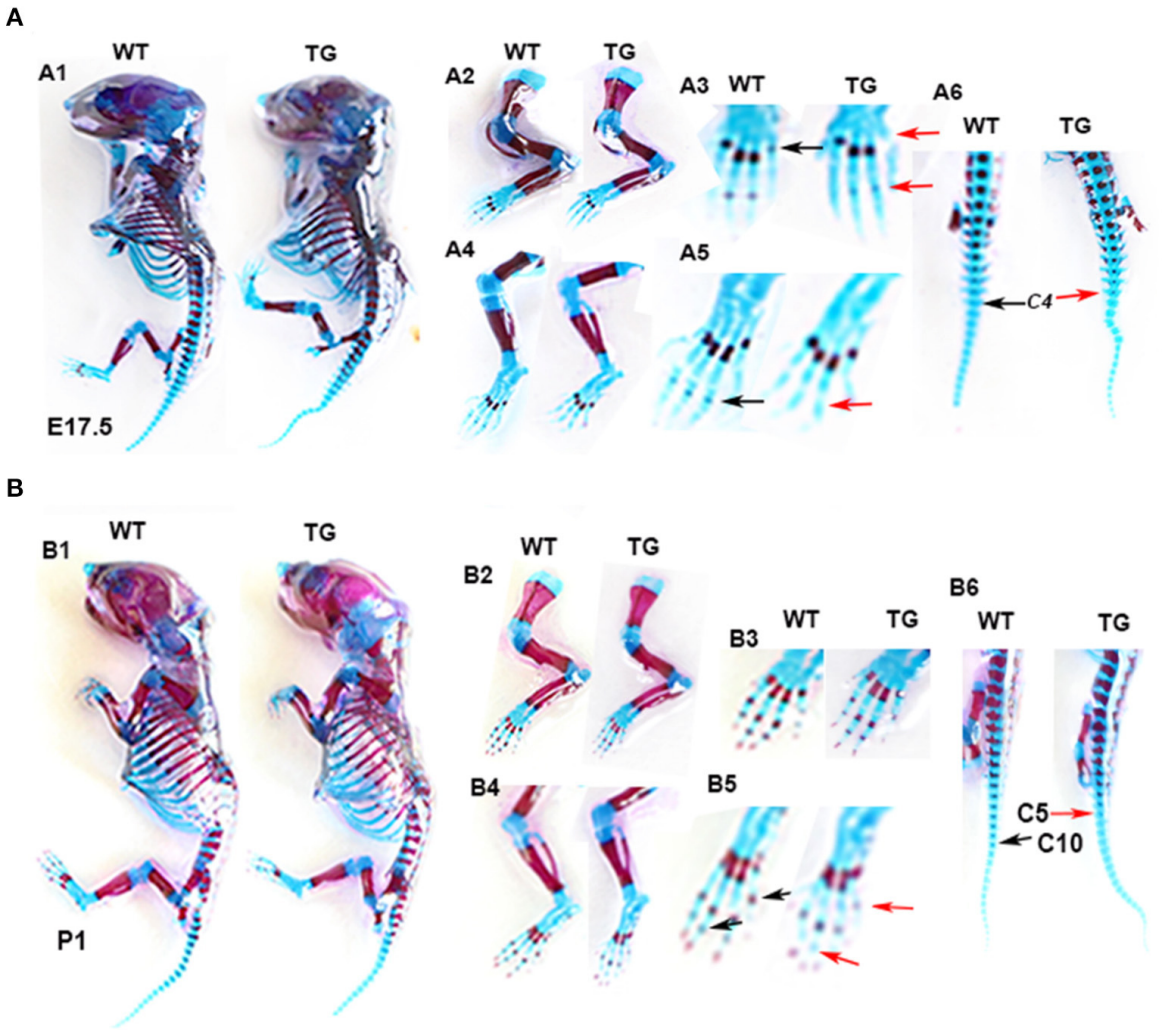
Skeletal phenotype of *Col10a1-Tbx5* TG mice. **(A)** Alcian blue and Alizarin red staining of the mouse skeleton on E17.5. A1: whole skeleton, no difference in Alizarin red staining between TG and WT mice. A2/A3 (zoomed-in pictures of A2): forelimb, no Alizarin red staining in the last phalange of a TG mouse (red arrow), but slight staining in a WT mouse (black arrow). A4/A5 (zoomed-in pictures of A4): hind limb, less Alizarin red staining in the phalanx of a TG mouse (red arrow) compared with a WT mouse (black arrow). **(B)** Alcian blue and Alizarin red staining of the mouse skeleton on P1. B1: whole skeleton, no difference in Alizarin red staining between TG and WT mice. B2/B3 (zoomed-in pictures of B2): forelimb, no difference between TG and WT mice. B4/B5 (zoomed-in pictures of B4): hind limb, less Alizarin red staining in the metatarsal bones and terminal digits of a TG mouse (red arrows) compared with a WT mouse (black arrows).

To determine the effect of Tbx5 overexpression on chondrogenic differentiation and mineralization in *ColX-Tbx5* TG mice, we performed whole-skeletal Alcian blue and Alizarin red staining in TG and WT mice on E17.5 and P1. Skeleton size and morphology were similar between TG and WT mice (Figure 8-A1, B1). Compared with WT mice, however, TG mice showed slightly weaker Alizarin red staining in the limb digits on E17.5 (Figure 8-A3, A5) and distal toe bones on P1 (Figure 8-B3, B5), indicating that *Tbx5* may play a limited role in late stages of endochondral ossification *in vivo*.

## Discussion

### Cell-Specific Expression of *Col10a1* Regulated by Its Cis-Enhancer Elements and Their Binding Factors

Several studies have identified multiple TFs and their binding sites that regulate the expression of *Col10a1* specifically in hypertrophic chondrocytes(Riemer et al., 2002; Adams et al., 2003; Schipani and Provot, 2003; Dong et al., 2005; Ijiri et al., 2005; Magee et al., 2005; Arnold et al., 2007; Dy et al., 2012; Maruyama et al., 2013). In particular, we found that Runx2 interacts with the *Col10a1* proximal promoter and its 150-bp cis-enhancer and contributes to its cell-specific expression *in vivo* (Zheng et al., 2003, 2009; Li et al., 2011). However, we also found that Runx2 interaction with this cis-enhancer is required but not sufficient for its reporter activity, suggesting that additional *Col10a1* regulators are required. In the present study, we identified 48 potential binding sites for ~40 candidate TFs using the TRAP program. Intriguingly, *in silico* sequence analysis of the *Col10a1* cis-enhancer indicates that multiple potential *Col10a1* transactivators (e.g., Gklf, Gli) and repressors (e.g., Tbx5) bind in the same location or adjacent to the Runx2 site (Gu et al., 2014), indicating that multiple TFs may regulate *Col10a1* expression and chondrocyte hypertrophy in skeletal development and disease.

### Identification of Candidate TFs in Mouse Chondrogenic Cell Models

Using binding affinity to predict candidate TFs can substantially increase the efficiency of experiments (Sandelin, 2008). Theoretically, genes predicted to interact with the specific enhancer of *Col10a1* should impact *Col10a1* expression and chondrocyte hypertrophy. Indeed, it was previously reported that interruption of Mef2c in cartilage delays hypertrophic differentiation in mouse endochondral bones, whereas its ectopic activation causes pre-mature hypertrophy (Arnold et al., 2007). Studies of craniofacial development show that Mef2c is required for normal expression of *Dlx5* in branchial arches. Notably, we detected significant upregulation of *Mef2c* and *Dlx5* mRNA levels in hypertrophic chondrogenic cell models which also show increased Col10a1 expression, suggesting a role of Dlx5 and Mef2c in chondrogenic differentiation. To determine their correlation with *Col10a1* expression and possible influence on endochondral ossification, we systematically examined the mRNA and protein levels of candidate TFs identified by the TRAP program in multiple mouse chondrogenic cell models. We found that the expression of most selected candidate genes was upregulated in hypertrophic MCT cells, ATDC5 cells, and mouse ribs. However, differences in the expression of candidate genes across models also existed, possibly due to differences in the tissues or cell populations selected and the corresponding approaches to analysis.

### *Tbx5* Repression of *Col10a1* Expression in Endochondral Ossification

The T-box gene family, which share a highly conserved 180 amino acid T-box DNA binding domain, may play essential roles in embryogenesis and cardiac development. In particular, Tbx5 has been extensively studied over the past decade because its mutation is associated with cardiac and limb defects observed in Holt-Oram syndrome (Mori and Bruneau, 2004; Steimle and Moskowitz, 2017). Notably, Tbx5 and Tbx4 are the earliest required factors for initiating hind and forelimb outgrowth, respectively. Tbx5 and Tbx4 directly regulate the expression of *Fgf10* and may establish a FGF signaling loop that drives successful limb outgrowth (Naiche and Papaioannou, 2003). By performing bioinformatics prediction of TFBSs for the *Col10a1* cis-enhancer, we found that Tbx5 binds to a sequence similar to the previously identified Runx2 binding site.

To determine the *in vivo* relevance of the *Tbx5* gene to *Col10a1* expression and chondrocyte hypertrophy, we performed RT-PCR, western blot, fluorescence immunohistochemistry, and immunohistochemistry of *Tbx5* to examine its expression in multiple chondrogenic differentiation cell models. We found that *Tbx5* expression is inversely correlated with *Col10a1* expression in chondrogenic cell models, suggesting that Tbx5 is a *Col10a1* transcriptional repressor. Overexpression of *Tbx5* in proliferative MCT cells downregulated the mRNA level of *Col10a1*, whereas knocking down *Tbx5* upregulated *Col10a1* expression. We also examined *Col10a1* expression in a stable *Tbx5*-overexpressing ATDC5 cell line. Previous studies show that ITS-induced ATDC5 cells, a model of endochondral ossification, show mature hypertrophy and upregulation of *Col10a1* (Newton et al., 2012). In the present study, *Tbx5* overexpression decreased *Col10a1* expression as early as day 7 in culture. Slightly weaker ALP staining was also observed on day 7, suggesting that Tbx5 negatively regulates mineralization during chondrocyte maturation.

To explore whether Tbx5 plays a similar role in regulating *Col10a1* expression *in vivo*, we generated *Col10a1-Tbx5* TG mice using a cell-specific *Col10a1* control element containing a 265-bp shorter basal promoter and four copies of a 300-bp cis-enhancer, which was previously shown to drive reporter (*LacZ*) gene expression in hypertrophic cells (Zheng et al., 2009). Skeletal staining showed slightly delayed ossification in the distal appendicular skeleton of TG mice compared with their WT littermates, suggesting that overexpression of *Tbx5* represses *Col10a1* expression *in vivo* and has a mild impact on skeletal ossification in mice. Additional studies are needed to elucidate how Tbx5 regulates skeletal development. Interestingly, Karouzakis et al. (2014) found that demethylation of the *Tbx5* promoter in rheumatoid arthritis synovial fibroblasts and synovium is associated with higher Tbx5 expression than in OA synovial fibroblasts and synovium. Han et al. (2019) also found that *Tbx5* is hypermethylated in OA patients. In addition, abnormal *COL10A1* expression and chondrocyte hypertrophy are observed in elderly OA patients. Combined with the present results, these findings suggest that Tbx5 may play a role in the progression of OA by impacting *Col10a1* expression and chondrocyte hypertrophy.

### Candidate TFs Regulate the Expression of *Col10a1* in Endochondral Ossification

Runx2 expression is mainly observed in the late condensation stage of chondrogenesis, substantially declines in proliferating chondrocytes, and reappears in pre-hypertrophic and hypertrophic chondrocytes (Kronenberg, 2003). We and others show that Runx2 interaction with a cis-enhancer element of *Col10a1* is required but not sufficient for *Col10a1* expression and contributes to its cell-specific expression across species. Indeed, multiple TFs regulate *Col10a1* expression, including Mef2c, Sox9, and Dlx5. Dlx5/6 and Mef2 form an enhanceosome with Tcf7, Ctnnb1, Sox5/6, Smad1, and Sp7 via protein-protein interactions, which activates the *Runx2* enhancer and affects *Co10a1* expression and chondrocyte hypertrophy (Komori, 2017).

In the present study, we identified many candidate *Col10a1* regulators through bioinformatics analysis of the mouse *Col10a1* enhancer. We also performed expression analysis to identify candidate TFs that positively (e.g., Sox17, Dlx5) and negatively (e.g., Tbx5) regulate *Col10a1* expression in different cell models. Furthermore, we found that *Tbx5* overexpression decreased *Col10a1* expression and resulted in slightly weaker ALP staining as early as day 7 of culture. Tbx5 has been shown to bind the NuRD complex by interacting with CHD4 and recruits it to regulatory regions containing T-box binding elements. The NuRD complex then deacetylates histones and remodels chromatin to a transcriptionally inactive state, thereby repressing target gene expression (Boogerd and Evans, 2016; Waldron et al., 2016; Zhu et al., 2017). As Tbx5 shows three putative binding sites on the 150-bp *Col10a1* cis-enhancer, we speculate that Tbx5 may directly interact with the *Col10a1* enhancer to regulate *Col10a1* expression.

Together, our results suggest that multiple TFs, including transactivators, inhibitors, and enhanceosomes, work with Runx2 to regulate *Col10a1* expression and chondrocyte maturation. We speculate that *Col10a1* transactivators accelerate chondrocyte maturation and endochondral ossification and thus could contribute to degradation of articular cartilage as seen in OA. Conversely, inhibitors or insufficient transactivators may decrease *Col10a1* expression and delay chondrocyte maturation and thus contribute to low bone growth as seen in skeletal dysplasia, or less cartilage degradation as seen in *Runx2*^+/−^ mice of an OA mouse model (Zhu et al., 2017). Further characterization of these candidate *Col10a1* regulators will open new avenues of research that aims to better understand skeletal developmental and disease and thus better options developing new therapeutic targets for skeletal diseases.

## Materials and Methods

### *In silico* Sequence Analysis of *Col10a1* Cis-Enhancer

The 150-bp *Col10a1* promoter/enhancer element (−4,196 to −4,147 bp) was subjected to *in silico* sequence analysis to search for transcription factor binding sites (TFBS) using following web-based softwares: TRAP (transcription factor affinity prediction) is a web tool to predict which TFs are susceptible to bind a promoter or genes of interest with highest affinity (Thomas-Chollier et al., 2011). TRAP uses the TRANSFAC database which was released in January, 2010. The search result is listed in a table ranking the affinity of TFs with a *p*-value (Supplementary Figure 1). TRAP is available online at http://trap.molgen.mpg.de/cgi-bin/home.cgi.

### Cell Culture, Total RNA Extraction and cDNA Synthesis

Mouse chondrocytes (MCT cells) were cultured at 32°C in standard DMEM with 8% FBS (Gibco, New Zealand) and 8% CO_2_ as per published protocol (Lefebvre et al., 1995; Shukunami et al., 1997). After grown until sub-confluence, these MCT cells were further cultured at either 32°C (proliferative) or 37°C for additional 3 days (become hypertrophic) before harvest. Total RNA was extracted using Trizol reagent (Invitrogen, Carlsbad, CA) according to the manufacturer's instructions. RNA was quantified using a NanoDrop spectrophotometer, and typically showed A260/280 ratios between 1.9 and 2.1. Total cDNA was synthesized by a commercial kit (iScript cDNA Synthesis kit, Bio-Rad, Hercules, CA) following the manufacturer's protocol. ATDC5 cells were maintained in a mixed DMEM/F-12 (1:1) medium (Invitrogen) with 5% FBS and 1% human insulin, transferrin, and sodium selenite (ITS, Sigma) at 37°C and 5% CO_2_ (Koshimizu et al., 2012). Cells were then harvested at days 0, 4, 7, 10, 14, and 21 and subjected to RNA extraction and cDNA synthesis respectively as described above.

### Expression Analysis of Genes Using Real-Time/qRT-PCR

The RT product was subjected to real-time or quantitative polymerase chain reaction (qRT-PCR) to show the relative mRNA levels of genes of interest. These genes include hypertrophic chondrocyte-specific *Col10a1*, 32 candidate *Col10a1* regulators and the endogenous control gene Gapdh for normalization of the RNA quality and quantity. For qRT-PCR, the cDNA templates were amplified with relevant gene- or 32 candidate regulators-specific primers (listed in Table 1) using the Bio-Rad iQ™ SYBR Green supermix and Bio-Rad CFX^TM^96 Detection System. Expression of selected genes was quantified by real-time PCR using an Applied Biosystems 2720 Fast Real-time PCR system (Applied Biosystems), following the manufacturer's instructions. The mean threshold cycle number (CT values) of target genes was normalized to endogenous Gapdh and calculated using 2^−^ΔΔCt and student *t*-test (Livak and Schmittgen, 2001; Pfaffl, 2001). Data was collected from multiple runs of real-time PCR with duplicate templates and the relative mRNA level was compared between proliferative and hypertrophic MCT cells, between day 0 and day 14 ATDC5 cells and between primary chondrocytes from the proliferative and hypertrophic zone by micro-dissection of mouse ribs. *p* < 0.05 was considered statistically significant fold change of mRNA level between samples.

### Western Blot Analysis

MCT cells (proliferative and hypertrophic) were washed with cold phosphate-buffered saline (PBS) and lyzed in RIPA buffer (Beyotime Biotechnology, CA, China) containing protease inhibitor cocktail (KangChen, Shanghai, China) for 30 min incubation on ice, and then centrifugated at 14,000 rpm for 10 min to remove cellular debris. The supernatant was collected and the protein concentration was determined by BCA-assay (Eppendorf, Hamburg, Germany). Then, 50 μg of total protein were subjected to SDS-PAGE and subsequently transferred onto Immobilon-P membranes (Millipore, Billerica, USA) which were then blocked with 5% non-fat milk for 1 h under continuous shaking. These membranes were then treated with different primary antibodies (goat anti-EGR2 1:1,000, goat anti-TBX5 1:1,000, goat anti-DLX5 1:1,000, goat anti-GKLF 1:1,000, goat anti-SRY 1:1,000, goat anti-COL10A1 1:1,000) (Santa Cruz Biotechnology, CA, USA) independently at 4°C overnight. These membranes were washed with TBST containing 0.1% Tween20 three times and then incubated with horseradish peroxidase conjugated rabbit anti-goat IgG antibody (Fcmacs Biotechnology, CA, China) at room temperature for 1 h. Specific bands were detected by an enhanced chemiluminescence system (Minichemi, China). Anti-actin was used to ensure equal loading by scanning densitometric analysis of the X-ray films. Western blot assay was performed in triplicate.

### Immunohistochemistry (IHC) Analysis

Sagittal sections of mouse hind limbs at the age of 1 day were subjected to IHC analysis using different primary antibodies (goat anti-TBX5 1:100 and goat anti-COL10A1 1:100) (Santa Cruz Biotechnology, CA, USA). Briefly, paraffin-embedded limb sections undergone de-paraffin and rehydration were subjected to antigen retrieval by incubation with hot (95°C) sodium citrate buffer (0.01 M, pH 6.0) for 10 min. The tissue sections were then exposed to hydrogen peroxide (3% H_2_O_2_) for 5 min to quench the endogenous peroxidase, followed by blocking with 30% goat serum (30 min). The slides were incubated overnight with above primary antigen at 4°C. Non-immune goat IgG was used as a negative control. After washing with the 1xTBST (Tris Buffered Saline with 0.1% Tween-20), the slides were further incubated with biotinylated secondary antibody (anti-goat IgG, Santa Cruz, CA) and detected using the ABC kit (Elite PK-6200 Universal, VECTOR laboratories, Burlingame, CA).

### Fluorescence Immunohistochemistry

Ten micrometers frozen sagittal sections of mouse hind limbs at the age of 1 day were subjected to fluorescence immunohistochemistry analysis using different primary antibodies (goat anti-EGR2 1:1,000, goat anti-TBX5 1:100, goat anti-DLX5 1:100, goat anti-GKLF 1:100, goat anti-SRY 1:100, goat anti-COL10A1 1:100) (Santa Cruz Biotechnology, CA, USA). Sections washed three times with phosphate buffer (PBS, PH 7.4), and permeabilized with ice-cold 0.3% Triton X-100 for 10 min at room temperature (RT), and blocked in PBS containing 5% goat serum albumin (BSA) for 30 min at RT. The sections were incubated overnight at 4°C with above primary antigen. Non-immune goat IgG was used as a negative control. After washing with the PBS (Tris Buffered Saline with 0.1% Tween-20), the slides were further incubated with Alexa Fluor 488 – conjugated Affinipure Rabbit Anti- Goat IgG (1:200, Santa Cruz) for 1 h at room temperature. Nuclear counterstaining was performed with DAPI for 10 min at RT. Immunofluorescence images were acquired using A Zeiss fluorescence microscope with 20×.

### Transfection, Establishment of *Tbx5* Expressing Stable Cell Line

MCT cells grown in 6-well plates at 32°C and reached 70–80% confluence were used for transient transfection studies as previously described (Zheng et al., 2003; Gu et al., 2015). Specifically, 4 μg of Tbx5 expression plasmid (MR227369, Origene) with blank and control vector *pCMV6*-entry (PS100001, Origene, Rockville, MD, USA) were transfected respectively using serum-free medium and Lipofectamine-plus (GIBCO BRL). After transfection for 6 h, cells were switched to 37 °C and continually cultured for 24 h in complete medium. The small interfering RNA (siRNA) sequences targeting *Tbx5* and scramble control sequence were purchased from Origene Technologies. Transfection of siRNAs and the scrambled control duplex in MCT cells was performed using siTran 1.0 reagent (Origene) according to the manufacturer's instructions. To establish the Tbx5 expressing stable cell line, ATDC5 cells grown in 37°C were transfected with Tbx5 expressing plasmid (*pCMV6-Tbx5* and *Colx-Tbx5*) or *pCMV6*-entry as a control while reached 70–80% confluence using similar procedures as described above. Cells were then cultured in DMEM/F12 medium containing 5% FBS and neomycin G418 (600 μg/ml, 158782, MP Biomedicals). After G418 selection for 2 weeks, three colonies were picked up from the survival colonies that were confirmed to have integrated with *Tbx5* expression plasmid and used for subsequent experiments.

### Generation of *Col10a1*–*Tbx5* Transgenic Mice

To generate the additional transgenic mice, a recombinant 3.9kb fragment with the hypertrophic chondrocyte-specific *Col10a1* regulatory elements as previously described followed by the Flag-tagged mouse *Tbx5* cDNA and a polyA sequence released from a *pCMV6-Tbx5* expression plasmid (MR227369, Origene) was used for DNA microinjection. Specifically, the *Col10a1* regulatory elements containing four copies of the 288-bp *Col10a1* cis-enhancer (4,296 to −4,209 bp) and a short *Col10a1* basal promoter (−220 to +45 bp) were released from plasmid *PBS-4x300-sh-XBP* by SpeI and SalI (blunted) digestion and cloned into *pCMV6-Tbx5* vector to replace the CMV promoter. Before microinjection, the *Col10a1–Tbx5* cassette digested from the recombinant vector by SpeI and PciI were purified by purification kit (Qiagen) and confirmed by sequencing. The transgenic mice were established in Nanjing Normal University by the research group of Professor Du. Briefly, the transgenic DNA construct was injected into pronuclei of ICR mouse zygotes and transplanted into pseudopregnant ICR mice. All the animal studies were approved by the animal care and oversight committees at Jiangsu University of Medicine.

### Alcian Blue, ALP, and Alizarin Red Staining

For Alcian blue staining, ATDC5 cells from Tbx5 stable line and controls undergoing differentiation were rinsed twice with PBS, and fixed with methanol for 2 min at −20°C. After fixation and rinse with PBS, cells were stained overnight with 0.1% Alcian blue (A0298-1g, Biotechnology, Shanghai, China) in 0.1 N HCL, followed by wash with distilled water and observation and image analysis under Nikon microscope (Japan). For Alkaline phosphatase (ALP) staining, ATDC5 cells were stained according to manufacturer's instruction (CAKP D001-2, Jiancheng, Biotechnology Company Ltd. Nanjing, China). Briefly, cells were washed twice with PBS and fixed with 4% paraformaldehyde for 3 min, followed by incubation with freshly prepared alkaline phosphatase substrate for 15 min at 37°C in a humidified dark box. Cells were washed with PBS and counter-stained with hematoxylin-eosin before microscopic analysis. For Alizarin red staining, cells were washed twice with PBS and fixed with 95% ethanol for 10 min before staining with 1% Alizarin red (A5333, Sigma, PH 6.4) for 10 min at room temperature and then for microscopic analysis. The staining intensity were measured by analyzing mean integrated optical density using the Image-Pro Plus 6.0 image analysis software (Media Cybernetics, Inc. Silver Spring, MD USA). The signal intensity of Alizarin red used for staining mineralized cartilaginous and bony matrices was applied to evaluate the ossification status of the mouse limb, digit, and tail bones (ossified caudal vertebrae numbers). Meanwhile, at least three lines of transgenic mice and wild type littermates were stained and analyzed for each developmental stage.

## Statistical Analysis

Expression of marker genes by qRT-PCR was analyzed using GraphPad prism 5 software. Relative mRNA levels of marker genes and Gapdh control were quantified by the comparative 2 –ΔΔCt method (Livak and Schmittgen, 2001). Date were collected from three repeated runs with duplicated templates and illustrated are results of representative runs. Analysis of variance (ANOVA) was used to compare between two or more groups. *p* < 0.05 implies significant fold changes of genes of interest in treated cells compared with controls.

## Data Availability Statement

The original contributions presented in the study are included in the article/Supplementary Material, further inquiries can be directed to the corresponding authors.

## Ethics Statement

The animal study was reviewed and approved by animal care and oversight committees at Jiangsu University School of Medicine.

## Author Contributions

QZ and LQ: conception, design, collection, and assembly of data. HB, TZ, and YLi: provision of study materials. RH, XZ, XL, JC, YLu, and JG: data analysis and interpretation. All authors: manuscript writing and final approval of manuscript.

## Conflict of Interest

YLu and QZ were employed by company Shenzhen Academy of Peptide Targeting Technology at Pingshan and Shenzhen Tyercan Bio-pharm Co., Ltd., Shenzhen, China. The remaining authors declare that the research was conducted in the absence of any commercial or financial relationships that could be construed as a potential conflict of interest.

## Supplementary Material

Supplementary Figure 1 Affinity-based ranking of TFBSs for the 150-bp *Col10a1* cis-enhancer. The 150-bp *Col10a1* promoter/enhancer element (−4,196 to −4,147 bp) was subjected to *in silico* sequence analysis to search for transcription factor binding sites (TFBSs) using TRAP. The search result is listed in a table ranking the affinity of TFs with a *p*-value.

Supplementary Figure 2 Effects of Tbx5 on cartilage matrix within chondrogenic differentiation in ATDC5 cells. Cells cultured for 7 days showed the strongest Alcian blue staining, but there were no differences between *Tbx5*-overexpressing and control cells across days.

## References

[B1] Adams S. L. Pallante K. M. Niu Z. Cohen A. J. Lu J. LeBoy P. S. (2003). Stimulation of type-X collagen gene transcription by retinoids occurs in part through the BMP signaling pathway. J. Bone Joint Surg. Am. 85-A(Suppl. 3), 29–33. 10.2106/00004623-200300003-00006 12925606

[B2] Ain N. U. Makitie O. Naz S. (2018). Autosomal recessive chondrodysplasia with severe short stature caused by a biallelic COL10A1 variant. J. Med. Genet. 55, 403–407. 10.1136/jmedgenet-2017-104885 28830906

[B3] Armiento A. R. Alini M. Stoddart M. J. (2019). Articular fibrocartilage - why does hyaline cartilage fail to repair? Adv. Drug Deliv. Rev. 146, 289–305. 10.1016/j.addr.2018.12.015 30605736

[B4] Arnold M. A. Kim Y. Czubryt M. P. Phan D. McAnally J. Qi X.. (2007). MEF2C transcription factor controls chondrocyte hypertrophy and bone development. Dev. Cell 12, 377–389. 10.1016/j.devcel.2007.02.004 17336904

[B5] Bateman J. F. Wilson R. Freddi S. Lamandé S. R. Savarirayan R. (2005). Mutations of COL10A1 in schmid metaphyseal chondrodysplasia. Hum. Mutat. 25, 525–534. 10.1002/humu.20183 15880705

[B6] Boogerd C. J. Evans S. M. (2016). TBX5 and NuRD divide the heart. Dev. Cell 36, 242–244. 10.1016/j.devcel.2016.01.015 26859347 PMC5542051

[B7] Coghlan R. F. Oberdorf J. A. Sienko S. Aiona M. D. Boston B. A. Connelly K. J.. (2017). A degradation fragment of type X collagen is a real-time marker for bone growth velocity. Sci. Transl. Med. 9:eaan4669. 10.1126/scitranslmed.aan4669 29212713 PMC6516194

[B8] Debiais-Thibaud M. Simion P. Ventéo S. Muñoz D. Marcellini S. Mazan S.. (2019). Skeletal mineralization in association with type X collagen expression is an ancestral feature for jawed vertebrates. Mol. Biol. Evol. 36, 2265–2276. 10.1093/molbev/msz145 31270539 PMC6759074

[B9] Dong Y. Drissi H. Chen M. Chen D. Zuscik M. J. Schwarz E. M.. (2005). Wnt-mediated regulation of chondrocyte maturation: modulation by TGF-beta. J. Cell Biochem. 95, 1057–1068. 10.1002/jcb.20466 15962307 PMC2649667

[B10] Drissi H. Zuscik M. Rosier R. O'Keefe R. (2005). Transcriptional regulation of chondrocyte maturation: potential involvement of transcription factors in OA pathogenesis. Mol. Aspects Med. 26, 169–179. 10.1016/j.mam.2005.01.003 15811433

[B11] Drissi M. H. Li X. Sheu T. J. Zuscik M. J. Schwarz E. M. Puzas J. E.. (2003). Runx2/Cbfa1 stimulation by retinoic acid is potentiated by BMP2 signaling through interaction with Smad1 on the collagen X promoter in chondrocytes. J. Cell Biochem. 90, 1287–1298. 10.1002/jcb.10677 14635200

[B12] Dy P. Wang W. Bhattaram P. Wang Q. Wang L. Ballock R. T.. (2012). Sox9 directs hypertrophic maturation and blocks osteoblast differentiation of growth plate chondrocytes. Dev. Cell 22, 597–609. 10.1016/j.devcel.2011.12.024 22421045 PMC3306603

[B13] Girkontaite I. Frischholz S. Lammi P. Wagner K. Swoboda B. Aigner T.. (1996). Immunolocalization of type X collagen in normal fetal and adult osteoarthritic cartilage with monoclonal antibodies. Matrix Biol. 15, 231–238. 10.1016/S0945-053X(96)90114-6 8892223

[B14] Gratal P. Mediero A. Sánchez-Pernaute O. Prieto-Potin I. Lamuedra A. Herrero-Beaumont G.. (2019). Chondrocyte enlargement is a marker of osteoarthritis severity. Osteoarthr. Cartil. 27, 1229–1234. 10.1016/j.joca.2019.04.013 31051241

[B15] Grskovic I. Kutsch A. Frie C. Groma G. Stermann J. Schlotzer-Schrehardt U.. (2012). Depletion of annexin A5, annexin A6, and collagen X causes no gross changes in matrix vesicle-mediated mineralization, but lack of collagen X affects hematopoiesis and the Th1/Th2 response. J. Bone Miner. Res 27, 2399–2412. 10.1002/jbmr.1682 22692895

[B16] Gu J. Liang Y. Qiao L. Lu Y. Hu X. Luo D.. (2015). URI expression in cervical cancer cells is associated with higher invasion capacity and resistance to cisplatin. Am. J. Cancer Res. 5, 1353–1367. 26101702 PMC4473315

[B17] Gu J. Lu Y. Li F. Qiao L. Wang Q. Li N.. (2014). Identification and characterization of the novel Col10a1 regulatory mechanism during chondrocyte hypertrophic differentiation. Cell Death Dis. 5:e1469. 10.1038/cddis.2014.444 25321476 PMC4649528

[B18] Gu J. Lu Y. Qiao L. Ran D. Li N. Cao H.. (2013). Mouse p63 variants and chondrogenesis. Int. J. Clin Exp. Pathol. 6, 2872–2879. 24294373 PMC3843267

[B19] Han B. Zheng Z. Ren J. Qiu W. Li X. (2019). Analysis of methylation datasets identified significantly changed genes and functional pathways in osteoarthritis. Clin. Rheumatol. 38, 3529–3538. 10.1007/s10067-019-04700-4 31376087

[B20] He Y. Manon-Jensen T. Arendt-Nielsen L. Petersen K. K. Christiansen T. Samuels J.. (2019). Potential diagnostic value of a type X collagen neo-epitope biomarker for knee osteoarthritis. Osteoarthr. Cartil. 27, 611–620. 10.1016/j.joca.2019.01.001 30654118

[B21] Higashikawa A. Saito T. Ikeda T. Kamekura S. Kawamura N. Kan A.. (2009). Identification of the core element responsive to runt-related transcription factor 2 in the promoter of human type X collagen gene. Arthritis Rheum 60, 166–178. 10.1002/art.24243 19116917

[B22] Ijiri K. Zerbini L. F. Peng H. Correa R. G. Lu B. Walsh N.. (2005). A novel role for GADD45beta as a mediator of MMP-13 gene expression during chondrocyte terminal differentiation. J. Biol. Chem. 280, 38544–38555. 10.1074/jbc.M504202200 16144844 PMC3937966

[B23] Ikegawa S. Nishimura G. Nagai T. Hasegawa T. Ohashi H. Nakamura Y. (1998). Mutation of the type X collagen gene (COL10A1) causes spondylometaphyseal dysplasia. Am. J. Hum. Genet. 63, 1659–1662. 10.1086/302158 9837818 PMC1377637

[B24] Karouzakis E. Trenkmann M. Gay R. E. Michel B. A. Gay S. Neidhart M. (2014). Epigenome analysis reveals TBX5 as a novel transcription factor involved in the activation of rheumatoid arthritis synovial fibroblasts. J. Immunol. 193, 4945–4951. 10.4049/jimmunol.1400066 25320281

[B25] Kel A. E. Gossling E. Reuter I. Cheremushkin E. Kel-Margoulis O. V. Wingender E. (2003). MATCH: a tool for searching transcription factor binding sites in DNA sequences. Nucleic Acids Res. 31, 3576–3579. 10.1093/nar/gkg585 12824369 PMC169193

[B26] Komori T. (2017). Roles of Runx2 in skeletal development. Adv. Exp. Med. Biol. 962, 83–93. 10.1007/978-981-10-3233-2_6 28299652

[B27] Komori T. (2018). Runx2, an inducer of osteoblast and chondrocyte differentiation. Histochem. Cell Biol. 149, 313–323. 10.1007/s00418-018-1640-6 29356961

[B28] Komori T. Yagi H. Nomura S. Yamaguchi A. Sasaki K. Deguchi K.. (1997). Targeted disruption of Cbfa1 results in a complete lack of bone formation owing to maturational arrest of osteoblasts. Cell 89, 755–764. 10.1016/S0092-8674(00)80258-5 9182763

[B29] Koshimizu T. Kawai M. Kondou H. Tachikawa K. Sakai N. Ozono K.. (2012). Vinculin functions as regulator of chondrogenesis. J. Biol. Chem. 287, 15760–15775. 10.1074/jbc.M111.308072 22416133 PMC3346098

[B30] Kronenberg H. M. (2003). Developmental regulation of the growth plate. Nature 423, 332–336. 10.1038/nature01657 12748651

[B31] Lamas J. R. Rodriguez-Rodriguez L. Vigo A. G. Alvarez-Lafuente R. Lopez-Romero P. Marco F.. (2010). Large-scale gene expression in bone marrow mesenchymal stem cells: a putative role for COL10A1 in osteoarthritis. Ann. Rheum Dis. 69, 1880–1885. 10.1136/ard.2009.122564 20498197

[B32] Lee B. Thirunavukkarasu K. Zhou L. Pastore L. Baldini A. Hecht J.. (1997). Missense mutations abolishing DNA binding of the osteoblast-specific transcription factor OSF2/CBFA1 in cleidocranial dysplasia. Nat. Genet. 16, 307–310. 10.1038/ng0797-307 9207800

[B33] Lefebvre V. Garofalo S. de Crombrugghe B. (1995). Type X collagen gene expression in mouse chondrocytes immortalized by a temperature-sensitive simian virus 40 large tumor antigen. J. Cell Biol. 128, 239–245. 10.1083/jcb.128.1.239 7822418 PMC2120322

[B34] Li F. Lu Y. Ding M. Napierala D. Abbassi S. Chen Y.. (2011). Runx2 contributes to murine Col10a1 gene regulation through direct interaction with its cis-enhancer. J. Bone Miner. Res. 26, 2899–2910. 10.1002/jbmr.504 21887706 PMC3222790

[B35] Liao L. Zhang S. Zhou G. Q. Ye L. Huang J. Zhao L.. (2019). Deletion of Runx2 in condylar chondrocytes disrupts TMJ tissue homeostasis. J. Cell. Physiol. 234, 3436–3444. 10.1002/jcp.26761 30387127 PMC6318053

[B36] Linsenmayer T. F. Chen Q. A. Gibney E. Gordon M. K. Marchant J. K. Mayne R.. (1991). Collagen types IX and X in the developing chick tibiotarsus: analyses of mRNAs and proteins. Development 111, 191–196. 10.1242/dev.111.1.191 2015794

[B37] Livak K. J. Schmittgen T. D. (2001). Analysis of relative gene expression data using real-time quantitative PCR and the 2(-Delta Delta C[T]) Method. Methods 25, 402–408. 10.1006/meth.2001.1262 11846609

[B38] Lu Y. Qiao L. Lei G. Mira R. R. Gu J. Zheng Q. (2014). Col10a1 gene expression and chondrocyte hypertrophy during skeletal development and disease. Front. Biol. 9, 195–204. 10.1007/s11515-014-1310-6

[B39] Mackie E. J. Ahmed Y. A. Tatarczuch L. Chen K. S. Mirams M. (2008). Endochondral ossification: how cartilage is converted into bone in the developing skeleton. Int. J. Biochem. Cell Biol. 40, 46–62. 10.1016/j.biocel.2007.06.009 17659995

[B40] Magee C. Nurminskaya M. Faverman L. Galera P. Linsenmayer T. F. (2005). SP3/SP1 transcription activity regulates specific expression of collagen type X in hypertrophic chondrocytes. J. Biol. Chem. 280, 25331–25338. 10.1074/jbc.M412549200 15849196

[B41] Maruyama T. Miyamoto Y. Yamamoto G. Yamada A. Yoshimura K. Suzawa T.. (2013). Downregulation of carbonic anhydrase IX promotes Col10a1 expression in chondrocytes. PLoS ONE 8:e56984. 10.1371/journal.pone.0056984 23441228 PMC3575511

[B42] Mori A. D. Bruneau B. G. (2004). TBX5 mutations and congenital heart disease: Holt-Oram syndrome revealed. Curr. Opin. Cardiol. 19, 211–215. 10.1097/00001573-200405000-00004 15096952

[B43] Naiche L. A. Papaioannou V. E. (2003). Loss of Tbx4 blocks hindlimb development and affects vascularization and fusion of the allantois. Development 130, 2681–2693. 10.1242/dev.00504 12736212

[B44] Newton P. T. Staines K. A. Spevak L. Boskey A. L. Teixeira C. C. Macrae V. E.. (2012). Chondrogenic ATDC5 cells: an optimised model for rapid and physiological matrix mineralisation. Int. J. Mol. Med. 30, 1187–1193. 10.3892/ijmm.2012.1114 22941229 PMC3573767

[B45] Otto F. Thornell A. P. Crompton T. Denzel A. Gilmour K. C. Rosewell I. R.. (1997). Cbfa1, a candidate gene for cleidocranial dysplasia syndrome, is essential for osteoblast differentiation and bone development. Cell 89, 765–771. 10.1016/S0092-8674(00)80259-7 9182764

[B46] Pfaffl M. W. (2001). A new mathematical model for relative quantification in real-time RT-PCR. Nucleic Acids Res 29:e45. 10.1093/nar/29.9.e45 11328886 PMC55695

[B47] Qin X. Jiang Q. Nagano K. Moriishi T. Miyazaki T. Komori H.. (2020). Runx2 is essential for the transdifferentiation of chondrocytes into osteoblasts. PLoS Genet. 16:e1009169. 10.1371/journal.pgen.1009169 33253203 PMC7728394

[B48] Riemer S. Gebhard S. Beier F. Poschl E. von der Mark K. (2002). Role of c-fos in the regulation of type X collagen gene expression by PTH and PTHrP: localization of a PTH/PTHrP-responsive region in the human COL10A1 enhancer. J. Cell Biochem. 86, 688–699. 10.1002/jcb.10260 12210735

[B49] Saito T. Fukai A. Mabuchi A. Ikeda T. Yano F. Ohba S.. (2010). Transcriptional regulation of endochondral ossification by HIF-2alpha during skeletal growth and osteoarthritis development. Nat. Med. 16, 678–686. 10.1038/nm.2146 20495570

[B50] Sandelin A. (2008). Prediction of regulatory elements. Methods Mol. Biol. 453, 233–244. 10.1007/978-1-60327-429-6_11 18712306

[B51] Schipani E. Provot S. (2003). PTHrP, PTH, and the PTH/PTHrP receptor in endochondral bone development. Birth Defects Res. C Embryo Today 69, 352–362. 10.1002/bdrc.10028 14745975

[B52] Shen G. (2005). The role of type X collagen in facilitating and regulating endochondral ossification of articular cartilage. Orthod. Craniofac. Res. 8, 11–17. 10.1111/j.1601-6343.2004.00308.x 15667640

[B53] Shukunami C. Ishizeki K. Atsumi T. Ohta Y. Suzuki F. Hiraki Y. (1997). Cellular hypertrophy and calcification of embryonal carcinoma-derived chondrogenic cell line ATDC5 *in vitro*. J. Bone Miner. Res. 12, 1174–1188. 10.1359/jbmr.1997.12.8.1174 9258747

[B54] Simoes B. Conceicao N. Viegas C. S. Pinto J. P. Gavaia P. J. Hurst L. D.. (2006). Identification of a promoter element within the zebrafish colXalpha1 gene responsive to runx2 isoforms Osf2/Cbfa1 and til-1 but not to pebp2alphaA2. Calcif. Tissue Int. 79, 230–244. 10.1007/s00223-006-0111-6 17033725

[B55] Steimle J. D. Moskowitz I. P. (2017). TBX5: a key regulator of heart development. Curr. Top. Dev. Biol. 122, 195–221. 10.1016/bs.ctdb.2016.08.008 28057264 PMC5371404

[B56] Thomas-Chollier M. Hufton A. Heinig M. O'Keeffe S. Masri N. E. Roider H. G.. (2011). Transcription factor binding predictions using TRAP for the analysis of ChIP-seq data and regulatory SNPs. Nat. Protoc. 6, 1860–1869. 10.1038/nprot.2011.409 22051799

[B57] von der Mark K. Frischholz S. Aigner T. Beier F. Belke J. Erdmann S.. (1995). Upregulation of type X collagen expression in osteoarthritic cartilage. Acta Orthop. Scand. Suppl. 266, 125–129. 10.3109/17453679509157667 8553841

[B58] Waldron L. Steimle J. D. Greco T. M. Gomez N. C. Dorr K. M. Kweon J.. (2016). The cardiac TBX5 interactome reveals a chromatin remodeling network essential for cardiac septation. Dev. Cell 36, 262–275. 10.1016/j.devcel.2016.01.009 26859351 PMC4920128

[B59] Warman M. L. Abbott M. Apte S. S. Hefferon T. McIntosh I. Cohn D. H.. (1993). A type X collagen mutation causes Schmid metaphyseal chondrodysplasia. Nat. Genet. 5, 79–82. 10.1038/ng0993-79 8220429

[B60] Zheng Q. Keller B. Zhou G. Napierala D. Chen Y. Zabel B.. (2009). Localization of the cis-enhancer element for mouse type X collagen expression in hypertrophic chondrocytes *in vivo*. J. Bone Miner. Res. 24, 1022–1032. 10.1359/jbmr.081249 19113928 PMC2683646

[B61] Zheng Q. Zhou G. Morello R. Chen Y. Garcia-Rojas X. Lee B. (2003). Type X collagen gene regulation by Runx2 contributes directly to its hypertrophic chondrocyte-specific expression *in vivo*. J. Cell Biol. 162, 833–842. 10.1083/jcb.200211089 12952936 PMC2172833

[B62] Zhu T. Qiao L. Wang Q. Mi R. Chen J. Lu Y.. (2017). T-box family of transcription factor-TBX5, insights in development and disease. Am. J. Transl. Res. 9, 442–453. 28337273 PMC5340680

