# Supplementary figures and images for: Expression Profiling and Functional Analysis of Candidate Col10a1 Regulators Identified by the TRAP Program
Source: Front Genet. 2021 Jul 2;12:683939. doi: 10.3389/fgene.2021.683939 (PMC8283764; doi:10.3389/fgene.2021.683939)

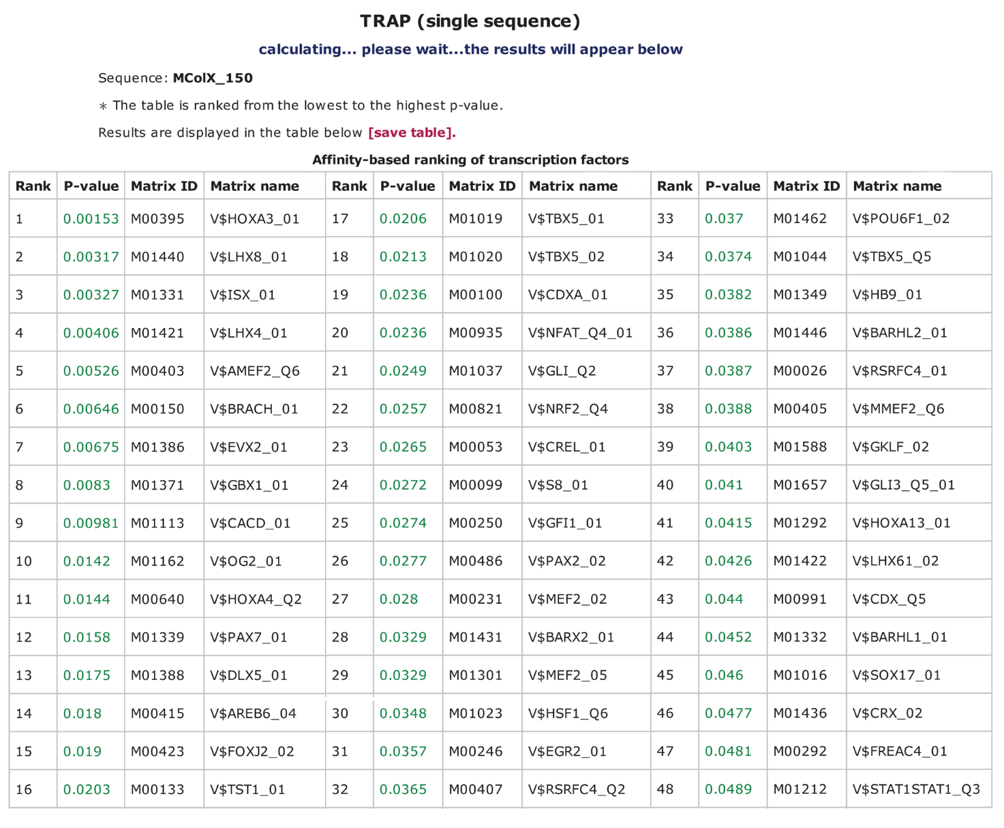

Supplement: Supplementary Figure 1 — Affinity-based ranking of TFBSs for the 150-bp Col10a1 cis-enhancer. The 150-bp Col10a1 promoter/enhancer element (−4,196 to −4,147 bp) was subjected to in silico sequence analysis to search for transcription factor binding sites (TFBSs) using TRAP. The search result is listed in a table ranking the affinity of TFs with a p-value. [file Image_1.TIF]

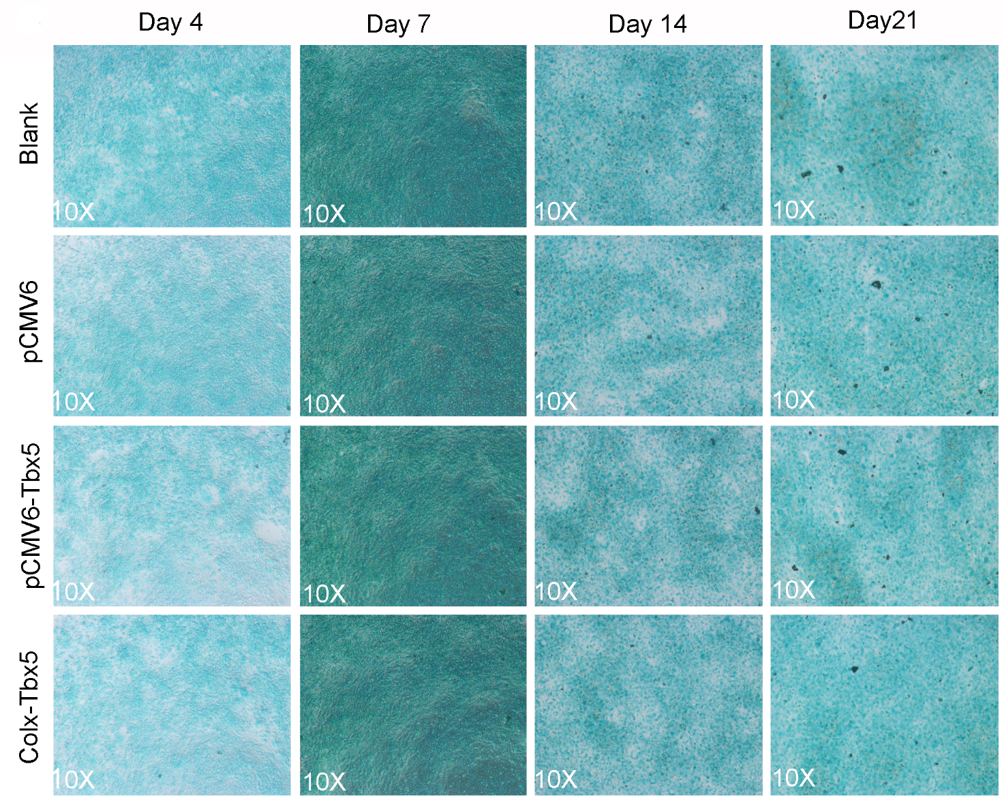

Supplement: Supplementary Figure 2 — Effects of Tbx5 on cartilage matrix within chondrogenic differentiation in ATDC5 cells. Cells cultured for 7 days showed the strongest Alcian blue staining, but there were no differences between Tbx5-overexpressing and control cells across days. [file Image_2.TIF]
